# Children with cerebral malaria or severe malarial anaemia lack immunity to distinct variant surface antigen subsets

**DOI:** 10.1038/s41598-018-24462-4

**Published:** 2018-04-19

**Authors:** Mark A. Travassos, Amadou Niangaly, Jason A. Bailey, Amed Ouattara, Drissa Coulibaly, Kirsten E. Lyke, Matthew B. Laurens, Jozelyn Pablo, Algis Jasinskas, Rie Nakajima, Andrea A. Berry, Matthew Adams, Christopher G. Jacob, Andrew Pike, Shannon Takala-Harrison, Li Liang, Bourema Kouriba, Abdoulaye K. Kone, J. Alexandra Rowe, JoAnn Moulds, Dapa A. Diallo, Ogobara K. Doumbo, Mahamadou A. Thera, Philip L. Felgner, Christopher V. Plowe

**Affiliations:** 1 Division of Malaria Research, Institute for Global Health, University of Maryland School of Medicine, Baltimore, Maryland United States of America; 2 Malaria Research and Training Center, University of Sciences, Techniques and Technologies of Bamako, Bamako, Mali; 3 Division of Infectious Diseases, Department of Medicine, University of California, Irvine, California, United States of America; 4 Wellcome Trust Sanger Institute, Hinxton, United Kingdom; 5 Centre for Immunity, Infection and Evolution, Institute of Immunology and Infection Research, School of Biological Sciences, University of Edinburgh, Edinburgh, United Kingdom; 6 LifeShare Blood Centers, Shreveport, Louisiana United States of America; 7 Duke Global Health Institute, Duke University, Durham, North Carolina United States of America

## Abstract

Variant surface antigens (VSAs) play a critical role in severe malaria pathogenesis. Defining gaps, or “lacunae”, in immunity to these *Plasmodium falciparum* antigens in children with severe malaria would improve our understanding of vulnerability to severe malaria and how protective immunity develops. Using a protein microarray with 179 antigen variants from three VSA families as well as more than 300 variants of three other blood stage *P. falciparum* antigens, reactivity was measured in sera from Malian children with cerebral malaria or severe malarial anaemia and age-matched controls. Sera from children with severe malaria recognized fewer extracellular PfEMP1 fragments and were less reactive to specific fragments compared to controls. Following recovery from severe malaria, convalescent sera had increased reactivity to certain non-CD36 binding PfEMP1s, but not other malaria antigens. Sera from children with severe malarial anaemia reacted to fewer VSAs than did sera from children with cerebral malaria, and both of these groups had lacunae in their seroreactivity profiles in common with children who had both cerebral malaria and severe malarial anaemia. This microarray-based approach may identify a subset of VSAs that could inform the development of a vaccine to prevent severe disease or a diagnostic test to predict at-risk children.

## Introduction

Malaria kills hundreds of thousands of children in sub-Saharan Africa annually. Two manifestations of severe malaria - cerebral malaria and severe malarial anaemia - account for most of these deaths^1^. The burden of severe malaria in this region falls principally on young children, particularly in areas of high malaria transmission^2^. In Bandiagara, Mali, the average age of a child with any form of severe malaria is between 21 and 39 months^3^. Episodes of cerebral malaria and severe malarial anaemia are rare after age four, while the median age of children with uncomplicated *P. falciparum* malaria is 10 years^4^. Thus, immunity to severe malaria appears to develop before the more common and less pathogenic uncomplicated malaria. The basis of natural immunity to severe malaria appears to be, at least in part, the acquisition of antibodies to parasite antigens expressed on the infected erythrocyte surface. In epidemiological studies, having antibodies to these parasite-produced erythrocyte variant surface antigens (VSAs) was consistently associated with protection against clinical malaria^5–8^.

The *var* family of genes encodes one group of VSAs: *P. falciparum* erythrocyte membrane protein-1 (PfEMP1) antigens, large molecules expressed on the surface of the infected erythrocyte^9^ that bind to endothelial receptors^10–16^. Extracellular regions of PfEMP1s protruding from erythrocytes and exposed to the immune system are comprised of constituent domains that include cysteine interdomain rich region (CIDR) domains and Duffy-binding like (DBL) domains. Each parasite genome carries a large repertoire of about 60 *var* genes, but each infected erythrocyte expresses only one PfEMP1 at a time. PfEMP1s mediate sequestration, a phenomenon whereby an infected red blood cell adheres to the endothelial membrane of an end-organ’s capillary wall, preventing its passage through the reticuloendothelial system and thus avoiding splenic clearance. Sequestration allows *P. falciparum* to evade the immune system and, in the brain, is likely critical to cerebral malaria pathogenesis.

The rapid acquisition of immunity to cerebral malaria in early childhood suggests that the lack of an immune response against a small subset of *var* genes could be responsible for this severe manifestation of malaria. In a case-control study of severe malaria conducted in Bandiagara, predominant expression of PfEMP1s that bind to the endothelial receptor CD36 was associated with hyperparasitemia, while expression of members of a distinct PfEMP1 group—which cannot bind to CD36 and have an as yet incompletely characterized set of targets—predominated in cerebral malaria episodes^17^. Non-CD36-binding PfEMP1s include subsets of PfEMP1s that have been associated with severe malaria^18–23^; VAR2CSA, which plays a primary role in pregnancy-associated malaria^24–26^; and conserved PfEMP1s encoded by *var1* and *var3*, which do not have well-characterized host binding sites^27,28^. The host binding sites of non-CD36-binding PfEMP1s have not been completely characterized. Some of these PfEMP1s bind endothelial protein C receptor (EPCR), blocking normal activity of protein C to regulate inflammation and coagulation^23^. The PfEMP1 CIDRα1 domain has been implicated in this pathway, binding to EPCR. In addition, a subset of these PfEMP1s bind both EPCR and intercellular adhesion molecule 1 (ICAM-1) and is particularly expressed in cerebral malaria^19,22^. The potential protective role of antibodies directed against the constitutive CIDR or DBL domains of these PfEMP1s is unknown.

Severe malarial anaemia studies also suggest that a smaller subset of *var* genes may contribute to pathogenesis and that the development of immunity is rapid. Expression of subsets of *var* genes encoding non-CD36-binding PfEMP1s has been associated with severe malarial anaemia^21,29^. Mathematical modelling has suggested that immunity to non-cerebral severe malaria such as severe malarial anaemia should develop after just one or two infections^30^.

We designed a protein microarray populated with fragments of variant surface antigens from the 3D7 reference genome, including PfEMP1 variants that have many of the commonly occurring consecutive *var* domain subtype motifs known as domain cassettes (DCs)^31^ and the much smaller STEVORs and RIFINs, which are encoded by the *repetitive interspersed family* (*rif)* and the *sub-telomeric variable open reading frame (stevor)* gene families, respectively. The array was probed with sera from a case-control study of severe malaria in Bandiagara to test the hypothesis that low seroreactivity to specific extracellular domains of PfEMP1 field variants is associated with vulnerability to cerebral malaria or to severe malarial anaemia. We predicted that severe malaria cases would have lower seroreactivity to a subset of PfEMP1 protein fragments than matched healthy controls and controls with uncomplicated malaria, as well as the convalescent sera of these cases. Children with severe malaria could thus be said to have serological “lacunae”, defined as gaps in the antibody repertoire reflecting a lack of exposure and/or a lack of an effective antibody response to a particular PfEMP1 variant. Given the extraordinary diversity of PfEMP1s, allele-specific immune recognition is likely, whereas cross-reactivity may be present in serorecognition of less diverse antigens. Such serological lacunae would also be present with fragments from the RIFIN and STEVOR VSA families, but not consistently present in serological responses to protein fragments of malaria vaccine candidate blood stage antigens apical membrane 1 (AMA1), merozoite surface protein 1 (MSP1), or reticulocyte binding-like homologue protein (RH5). Our results demonstrated that variant surface antigen lacunae predominated across case-control and acute-convalescent comparisons, with gaps in PfEMP1 seroreactivity particularly suggestive of vulnerability to severe malaria.

## Results

### Clinical characteristics

From 2000 to 2003, 78 children with severe malaria were enrolled that met criteria for cerebral malaria (CM) and/or severe malarial anaemia (SMA), including 43 CM cases, 24 SMA cases, and 11 cases with both CM and SMA (referred to as CM + SMA cases) (Table 1). For most subjects, we obtained sera from matched controls who were either healthy or had uncomplicated malaria. The majority of enrolled children were of the Dogon ethnicity and had Haemoglobin type AA.

**Table 1:** Baseline characteristics of subjects.

	Cerebral malaria			Severe malarial anaemia			Both cerebral malaria and severe malarial anaemia		
	Cases (n = 43)	Uncomplicated malaria controls (n = 43)	Healthy controls (n = 40)	Cases (n = 24)	Uncomplicated malaria controls (n = 21)	Healthy controls (n = 21)	Cases (n = 11)	Uncomplicated malaria controls (n = 9)	Healthy controls (n = 11)
Number female (%)	18 (41.9%)	18 (41.9%)	15 (37.5%)	8 (33.3%)	11 (52.4%)	11 (52.4%)	9 (81.8%)	2 (22.2%)	8 (72.7%)
Age: mean + SD (months)	30.9 ± 18.6	34.4 ± 21.4	30.9 ± 18.7	26.0 ± 17.8	22.7 ± 11.3	25.9 ± 17.8	31.3 ± 15.9	32.6 ± 19.6	32.4 ± 16.1
Minimum/maximum	4.7–79.5	9.3–97.7	6.6–76.2	7.3–78.5	7.4–51.2	6.2–68.4	11.0–64.7	6.5–77.1	9.0–65.0
Ethnicity									
Dogon	38 (88.4%)	36 (83.7%)	33 (82.5%)	20 (83.3%)	20 (95.2%)	17 (81.0%)	10 (90.9%)	8 (88.9%)	11 (100%)
Non-Dogon	5 (11.6%)	7 (16.2%)	7 (17.5%)	4 (16.7%)	1 (4.8%)	4 (19.0%)	1 (9.1%)	1 (11.1%)	0
Haemoglobin status^1^									
Hb AA	35 (87.5%)	31 (75.6%)	30 (78.9%)	24 (100%)	17 (81.0%)	19 (90.5%)	8 (80.0%)	5 (55.6%)	7 (63.6%)
Hb AC	5 (12.5%)	9 (22.0%)	3 (7.9%)	0	3 (14.3%)	0 (4.8%)	1 (10.0%)	2 (22.2%)	2 (18.2%)
Hb AS	0	0	5 (13.2%)	0	1 (4.8%)	1 (4.8%)	1 (10.0%)	1 (11.1%)	2 (18.2%)
Hb CC	0	1 (2.4%)	0	0	0	1 (4.8%)	0	1 (11.1%)	0
Parasitemia: median (p/μL)	80,000	6,500	0	83,112.5	9,825	0	37,125	9,300	0
25^th^–75^th^ percentiles	14,662.5–549,487.5	1,937.5–19,525	—	17,600–520,781.25	2,850–28,725	—	15,337.5–96,750	1,025–30,675	—
Haemoglobin: mean ± SD, g/dL	8.2 ± 2.1	8.9 ± 1.8	10.3 ± 1.6	4.3 ± 0.8	8.9 ± 1.6	9.6 ± 1.6	4.0 ± 0.5	9.1 ± 1.4	11.1 ± 1.1
Minimum/maximum	5.1–12.7	5.3–13.0	6.9–13.4	2.0–5.0	6.4–12.2	6.2–13.0	2.6–4.6	7.0–10.7	9.4–12.6
Blantyre Coma Scale: mean	1.3	5	5	4.7	5	5	1.5	5	5
25^th^–75^th^ percentiles	1–2	5–5	5–5	5–5	5–5	5–5	1–2	5–5	5–5
Minimum/maximum	0–2	5–5	5–5	3–5	5–5	5–5	0–2	5–5	5–5
Mortality (%)	6 (14.0%)	0	0	6 (25%)	0	0	3 (27.3%)	0	0
Convalescent samples (n)	27	—	—	12	—	—	0	—	—

Hb, haemoglobin; SD, standard deviation.

^1^Haemoglobin status was not successfully determined for all subjects.

### Breadth of antibody response: recognition of protein fragments

**Figure 1:**
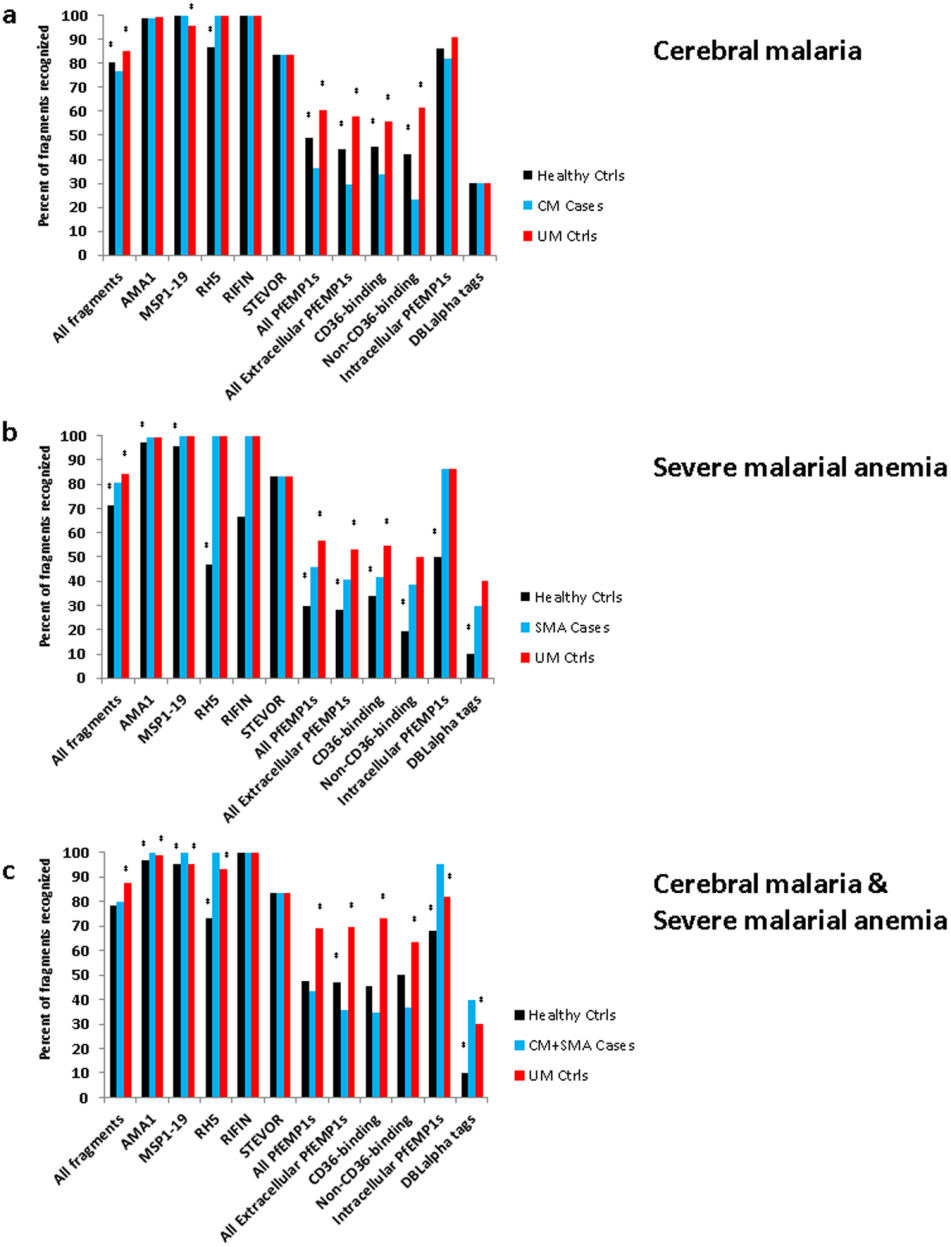
Breadth of recognition of *P. falciparum* antigens by study group. Percentage of recognized *P. falciparum* antigens for sera from each severe malaria group compared to their corresponding control groups. *Indicates a significant difference in percentage of recognized fragments between sera from a particular control group and the corresponding severe malaria group (P < 0.05, McNemar’s test). No statistical comparisons were made for RIFINs and STEVORs, as each of these VSA groups included fewer than 10 fragments.

To gauge overall levels of immunity, we determined how many *P. falciparum* fragments were recognized by sera of each subgroup compared to a negative control set of 11 malaria-naïve North American blood donor controls. The protein microarray included 170 PfEMP1 fragments, six full-length STEVOR antigens, and three full-length RIFIN antigens, all based on the 3D7 reference genome. We also studied merozoite surface-exposed blood stage antigens that exist as a single exon per genome but with varying levels of single-nucleotide polymorphisms that define antigen-specific parasite haplotypes. Specifically, the array contained 268 full-length AMA1s, the C-terminal 19 kDa portion of 20 merozoite surface protein 1s (MSP1-19), the entire 3D7 version of the MSP protein divided into two fragments, and 15 full-length RH5 proteins, all cloned from parasites isolated from field samples (data file S1). Similar to sera from their uncomplicated malaria and healthy controls, sera from CM cases recognized most of the AMA1, MSP1-19 and RH5 fragments (Fig. 1A). Both CM cases and controls recognized over 80 percent of the intracellular ATS domain PfEMP1 fragments. In contrast, sera from CM cases recognized less than a third of extracellular PfEMP1 fragments (29.7%), unlike uncomplicated malaria controls (58.0%) and healthy controls (44.25%). Convalescent sera from CM cases recognized fewer fragments than acute sera for all antigen groups analysed (Fig. S1A).

Sera from SMA cases and the uncomplicated malaria controls recognized most of the AMA1, MSP1-19, and RH5 fragments; however, sera from SMA cases recognized significantly fewer extracellular PfEMP1 fragments than uncomplicated malaria control sera did (Fig. 1B). In contrast, SMA sera recognized more protein fragments than healthy controls, including more AMA1, MSP1-19, RH5, extracellular PfEMP1s, and intracellular PfEMP1s. Overall, sera from convalescent SMA cases recognized fewer fragments than sera from acute SMA cases, but the two groups did not differ in recognition of intracellular or non-CD36-binding extracellular PfEMP1 fragments (Fig. S1B).

Sera from CM + SMA children recognized fewer extracellular PfEMP1 fragments than both uncomplicated malaria and healthy controls. Interestingly, sera from these cases recognized more intracellular PfEMP1s than either group of controls and also recognized more AMA1, MSP1-19, and RH5 fragments than either control group.

Sera from children with CM recognized fewer PfEMP1 fragments than children with CM + SMA or only SMA (Fig. S2). This included recognition of fewer non-CD36-binding extracellular PfEMP1 fragments and intracellular PfEMP1 fragments.

### Intensity of antibody response: identification of *falciparum* antigen lacunae

**Figure 2:**
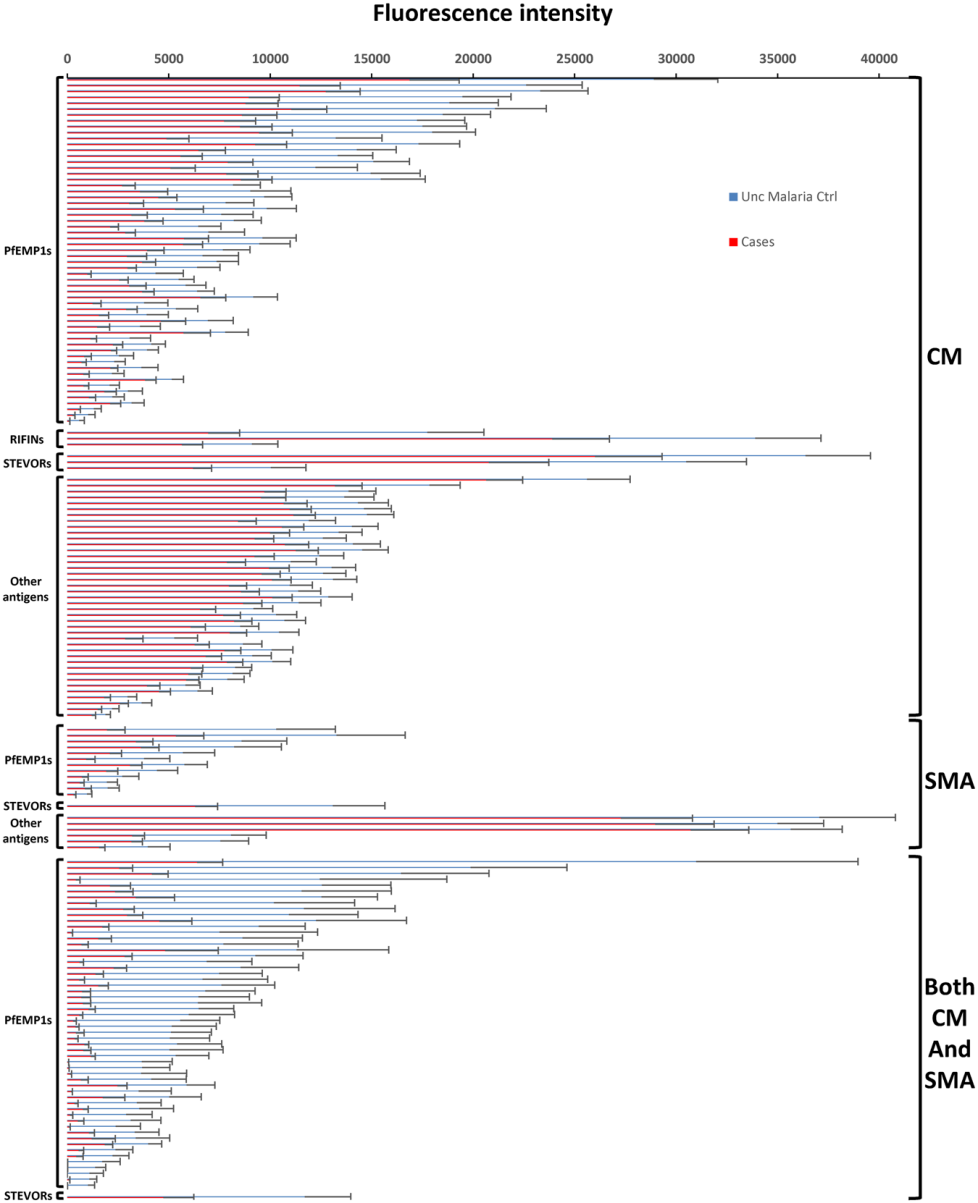
Differentially seroreactive *P. falciparum* antigens for sera from uncomplicated malaria controls versus severe malaria subjects. Sera from uncomplicated malaria controls reacted more intensely to 107, 19, and 57 fragments versus sera from subjects with cerebral malaria, severe malarial anaemia, and both cerebral malaria and severe malarial anaemia, respectively.

For the CM subgroup (n = 42), the sera of uncomplicated malaria controls reacted more intensely to 107 protein fragments than did sera of cases (Fig. 2; additional details in Fig. S3); for the CM + SMA subgroup (n = 9), we identified 57 such protein fragments. In contrast, for the SMA subgroup, the sera of uncomplicated malaria controls reacted more intensely to 19 protein fragments than did sera of cases (n = 21). In all three subgroups, the majority of these differentially reactive fragments were VSAs [CM cases: 65 of 107 fragments (60.7%); SMA cases: 13 of 19 fragments (68.4%); CM + SMA cases: 57 of 57 fragments (100%)].

AMA1, MSP1, and RH5 lacunae were not consistently present in severe malaria comparisons. Of 268 AMA1 variants, 39 elicited more intense responses by sera from uncomplicated malaria controls versus CM cases, while only 2 AMA1 variants were differentially seroreactive in the SMA subgroup. Of 15 RH5 fragments, two elicited more intense responses by sera from uncomplicated malaria controls versus CM cases; one RH5 fragment was differentially seroreactive in the SMA group. Sera from uncomplicated malaria controls did not react more intensely to any of the 22 MSP1 fragments compared to sera from CM cases; one MSP fragment was differentially seroreactive in the SMA group. No AMA1, RH5, or MSP1 lacunae were identified for CM + SMA children with respect to uncomplicated malaria controls.

#### Uncomplicated malaria control comparisons

Children with cerebral malaria possessed significant gaps in PfEMP1 immunity compared to uncomplicated malaria controls, including extracellular and intracellular fragment lacunae from both CD36-binding and non-CD36-binding PfEMP1s and both DBL-DBL and DBL-CIDR domain couplets. Differentially reactive CM subgroup fragments included PfEMP1s, STEVORs, and RIFINS (Fig. 2). Fifty-nine PfEMP1 fragments were differentially reactive in serologic comparisons between children with uncomplicated malaria versus cerebral malaria. Twenty-nine of these PfEMP1 fragments were from non-CD36-binding PfEMP1s; of 21 DBL-CIDR fragments, three were DBLα-CIDRα1 domain couplets. These non-CD36-binding PfEMP1s included all three 3D7 *var3-*encoded PfEMP1s. Twenty intracellular PfEMP1 domain lacunae were also identified, including seven intracellular non-CD36-binding PfEMP1s. Lacunae involving all three RIFINs and three of the six STEVORs were also present.

In contrast, children with severe malarial anaemia had comparatively fewer gaps in PfEMP1 immunity compared to their uncomplicated malaria controls. The 13 differentially reactive variant surface antigen fragments in the SMA analysis included 12 extracellular PfEMP1s and 1 STEVOR, but no RIFINs. PfEMP1 lacunae included six fragments from non-CD36-binding PfEMP1s overall, four DBL-DBL domain couplets (including two *var3-*encoded PfEMP1s), and eight DBL-CIDR domain couplets, including six DBLδ1-CIDRβ1 domain couplets, none of which were DBLα-CIDRα1 domain couplets.

Compared to their uncomplicated malaria controls, children with both cerebral malaria and severe malarial anaemia possessed similar numbers of PfEMP1 lacunae as children with only cerebral malaria. Differentially reactive CM + SMA subgroup fragments included 56 extracellular PfEMP1 fragments and 1 STEVOR. PfEMP1 lacunae included 19 fragments from non-CD36-binding PfEMP1s and a total of 35 DBL-CIDR domain couplets, including 15 DBL-CIDRα domain couplets, but no DBLα-CIDRα1 domain couplets. Three DBL-DBL couplets were from the *var1-*encoded PfEMP1 PFE1640w.

Lacunae that were shared between uncomplicated malaria control comparisons with CM, SMA, and CM + SMA cases were predominantly extracellular PfEMP1 fragments, but also included one STEVOR fragment (Table S1). Lacunae present in all three severe malaria subgroup comparisons included three extracellular PfEMP1 fragments and one STEVOR.

#### Healthy control comparisons

**Figure 3:**
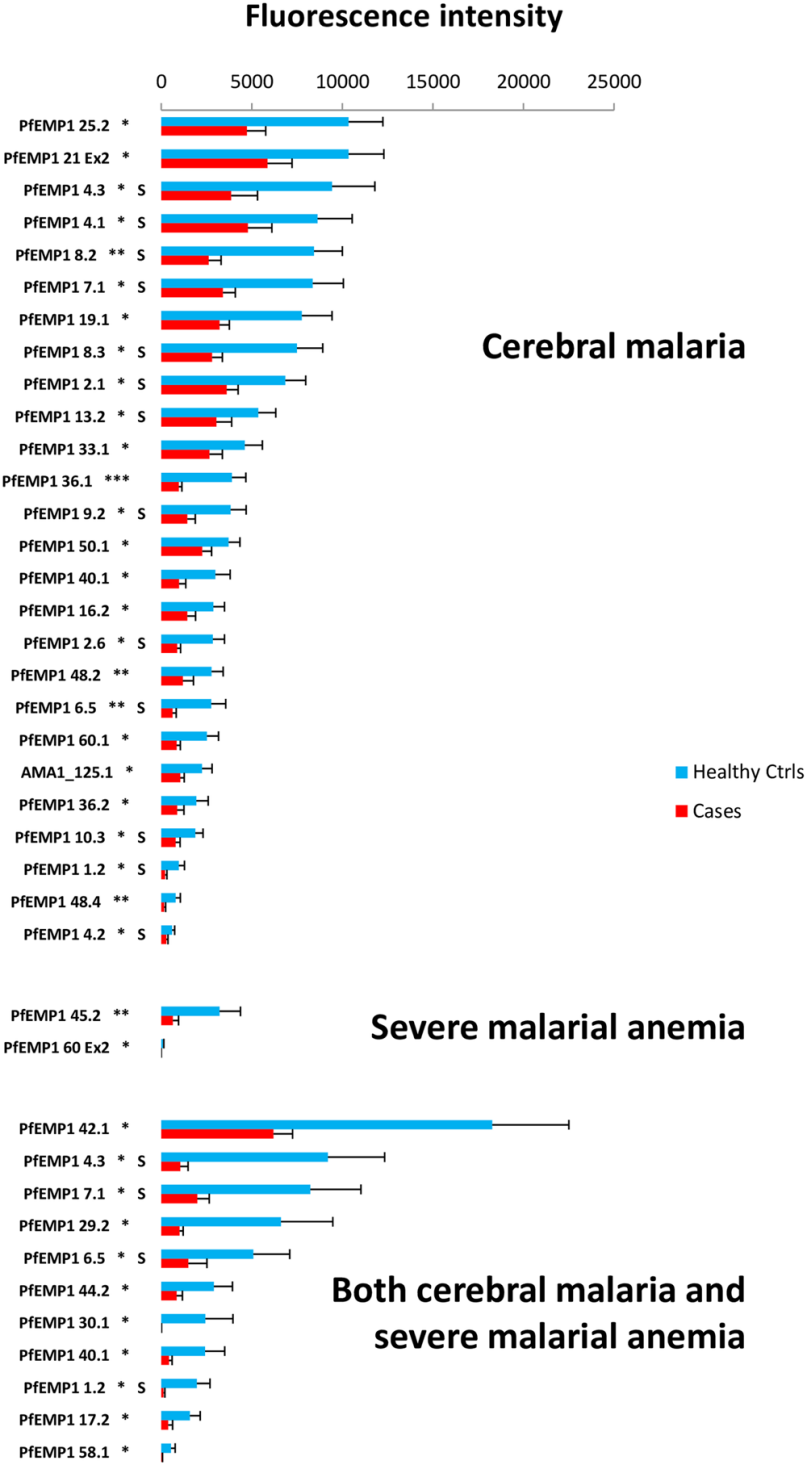
Differentially seroreactive *P. falciparum* antigens for sera from healthy controls versus severe malaria subjects. Sera from healthy controls reacted more intensely to 26, two, and 11 fragments versus sera from subjects with cerebral malaria, severe malarial anaemia, and both cerebral malaria and severe malarial anaemia, respectively. Non-CD36-binding PfEMP1 fragments are designated with an “S”. *Indicates P < 0.05; **P < 0.01, and ***P < 0.001 (Wilcoxon’s signed-rank test).

Comparisons between healthy controls and severe malaria cases revealed lacunae almost entirely associated with extracellular PfEMP1 fragments [37 of 39 fragments (94.9%)]. For CM case comparisons (n = 39), 26 lacunae were identified (Fig. 3), including 24 extracellular PfEMP1 fragments, one intracellular PfEMP1 fragment, and one AMA1. The majority of PfEMP1 fragments were from non-CD36-binding PfEMP1s (52.0%; 13 of 25 PfEMP1 fragments). Fourteen DBL-CIDR lacunae were identified, including eight DBLα-CIDRα, of which one was a DBLα-CIDRα1.

For SMA subjects (n = 21), only two lacunae were identified: one DBL-CIDR domain couplet and one intracellular PfEMP1 fragment, both from CD36-binding PfEMP1s.

For CM + SMA comparisons (n = 11), all 11 lacunae identified were from extracellular PfEMP1 fragments, four of which were non-CD36-binding PfEMP1s and were also found in the healthy control-CM comparison (listed below). Eight DBL-CIDR domain couplets were identified, none of which included DBLα-CIDRα1 domain couplets.

Only five lacunae were shared between healthy control comparisons with CM and CM + SMA cases, all of which were extracellular PfEMP1 fragments, including four non-CD36-binding PfEMP1 fragments (Table S2): a DBLβ-DBLγ domain couplet from the DC8-associated MAL6P1.316; the proximal portion of PF11_0008, a DC5 PfEMP1; the *var3*-encoded PfEMP1 PFA0015c; the proximal portion of PF13_0003, a DC16 PfEMP1; and the DC19 portion of the Group B PfEMP1 PFB1055c. Neither CM nor CM + SMA acute illness comparisons shared lacunae with SMA acute illness comparisons.

#### Convalescent sera comparisons

We sought to identify PfEMP1 fragments bound more intensely by sera following an episode of severe malaria than when a child was acutely ill. We were particularly interested in increased seroreactivity to CIDRα1-containing PfEMP1s. EPCR is a target of the CIDRα1 domain^23^. It is bound more readily by parasites from children with severe malaria than parasites from uncomplicated malaria^23^. CIDRα1-containing PfEMP1 fragments have been implicated in binding human brain endothelial cells *in vitro*^18,20^. Significant CIDRα1 expression has been observed in Tanzanian and Beninese children with cerebral malaria^21,32^ as has elevated expression of the non-CD36-binding PfEMP1s (a group that includes CIDRα1-containing PfEMP1 fragments) in cerebral malaria cases in Mali^17^ and Kenya^29^.

**Figure 4:**
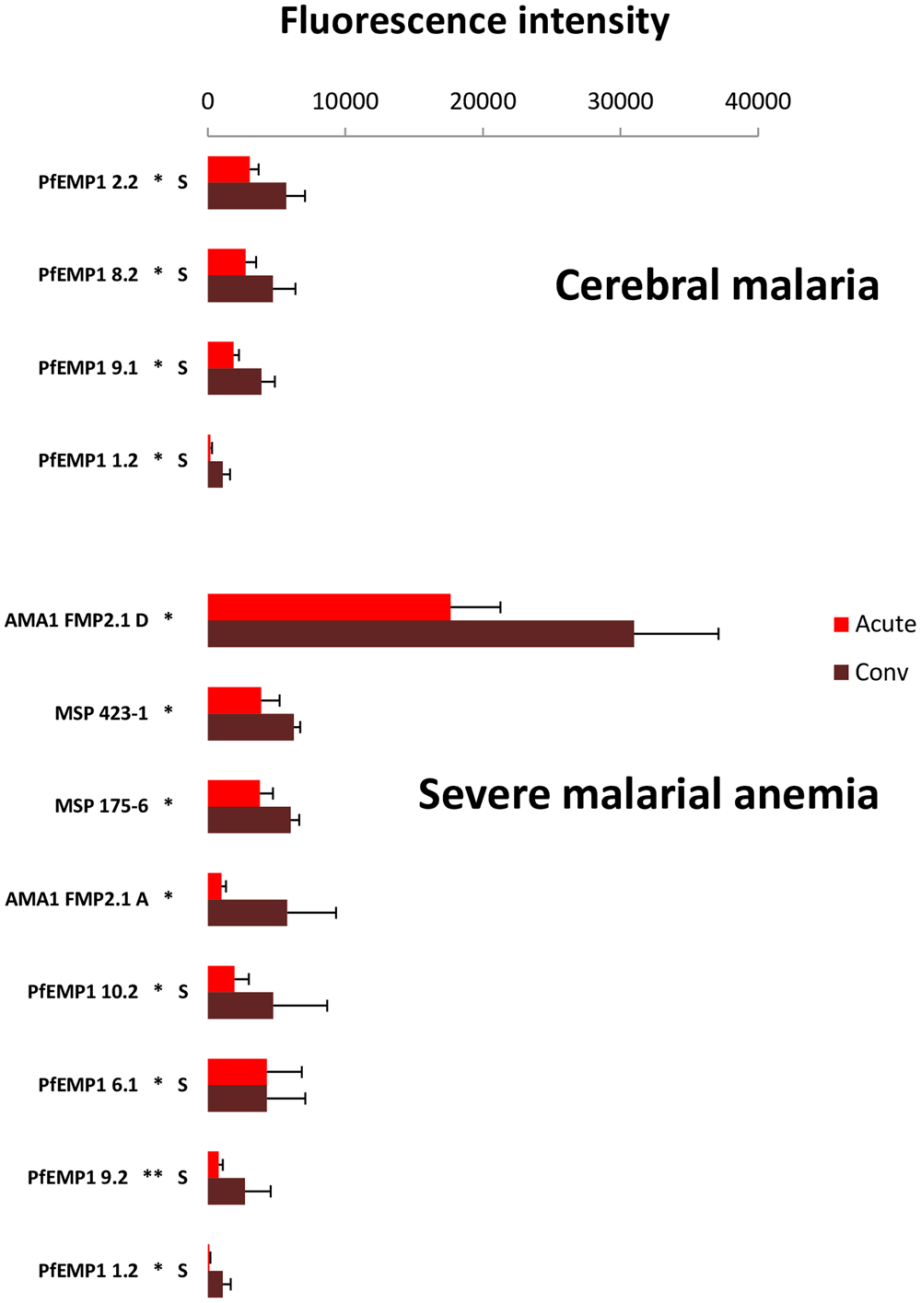
Differentially seroreactive *P. falciparum* antigens in convalescent versus acute sera comparisons for severe malaria subjects. Convalescent sera reacted more intensely to four and eight fragments versus acute sera of subjects with cerebral malaria and severe malarial anaemia, respectively. Non-CD36-binding PfEMP1 fragments are designated with an “S”. *Indicates P < 0.05; **P < 0.01, and ***P < 0.001 (Wilcoxon’s signed-rank test).

In comparisons between matched acute and convalescent sera from CM children, only four protein fragments, all from non-CD36-binding PfEMP1s containing proximal DBLα-CIDRα1 domain couplets, elicited more intense responses by convalescent sera than acute sera (Fig. 4; n = 27). Three of these fragments were DBLβ-DBLγ domain couplets associated with DC8^31^ (from PFD0020c, MAL6P1.316, and PF08_0140), and the fourth was a proximal DBLα-CIDRα1 domain couplet that is part of the DC4-containing PFD1235w, which binds both EPCR and ICAM-1^22^.

Eight protein fragments elicited more intense responses by convalescent sera than acutely ill sera from SMA children (n = 12). This included two AMA1 fragments, two MSP1-19 fragments, and four PfEMP1 fragments, all from non-CD36-binding PfEMP1s, including three with proximal DBLα-CIDRα1 domain couplets. These four PfEMP1 fragments included three DBL-DBL domain couplets (from the *var1*-encoded PFE1640w, the DC4-containing PFD1235w, and the DC8-associated MAL6P1.316) and one proximal DBLα-CIDRδ domain couplet, the DC16 portion of PF13_0003.

One PfEMP1 fragment was shared between CM and SMA convalescent comparisons – the non-CD36-binding fragment PfEMP1 1.2 (from MAL6P1.316); an additional non-CD36-binding PfEMP1, (PfEMP1 9; from PFD1235w), had different fragment lacunae identified for the CM comparison and for the SMA comparison (Table S3).

The limited number of convalescent samples prohibited meaningful comparison of acute versus convalescent sera of CM + SMA children (n = 2).

### Recurrent lacunae

For each severe malaria type, we compared seroreactivity from the group with acute disease with seroreactivities of the healthy control and uncomplicated malaria groups as well as with convalescent seroreactivity in order to identify lacunae that were present across multiple serology group comparisons.

#### Cerebral malaria

**Figure 5:**
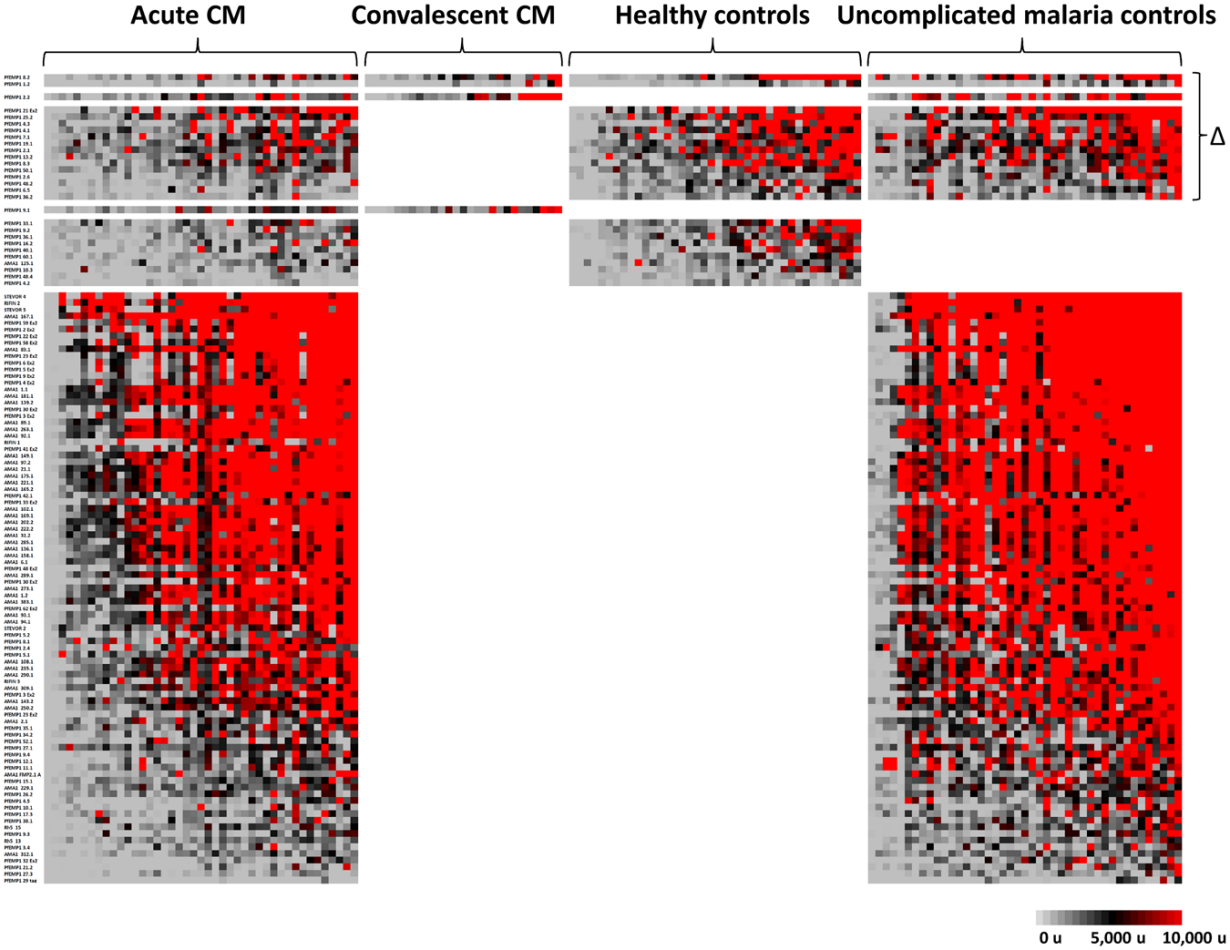
Heat map of lacunae shared across different acute cerebral malaria sera comparisons. *P. falciparum* antigen lacunae were grouped by recurrence across different comparisons with acute sera from cerebral malaria subjects. Each column displays the seroreactivity profile of one serum sample, and each row displays the seroreactivity profile of an individual protein fragment. Grey color indicates no seroreactivity, black is low-to-moderate seroreactivity, and red denotes high seroreactivity to probed fragments. Lacunae present across more than one comparison are designated by a triangle.

Seventeen lacunae were identified across more than one CM comparison (Fig. 5). All of these lacunae were PfEMP1 fragments, including 16 extracellular PfEMP1 fragments, 11 of which were non-CD36-binding PfEMP1 fragments. Two non-CD36-binding fragments, PfEMP1 8.2 and PfEMP1 1.2, were consistently identified as lacunae across all three acute CM sera comparisons and were DBLβ-DBLγ domain couplets from PFD0020c and MAL6P1.316, respectively, associated with DC8.

#### Severe malarial anaemia

**Figure 6:**
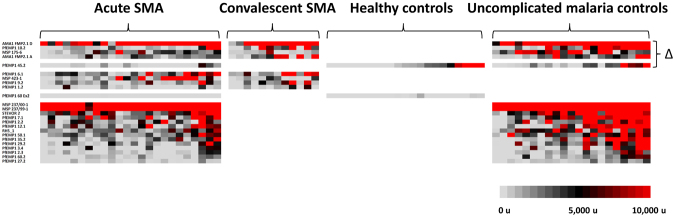
Heat map of lacunae shared across different acute severe malarial anaemia sera comparisons. *P. falciparum* antigen lacunae were grouped by recurrence across different comparisons with acute sera from severe malarial anaemia subjects. Each column displays the seroreactivity profile of one serum sample, and each row displays the seroreactivity profile of an individual protein fragment. Grey color indicates no seroreactivity, black is low-to-moderate seroreactivity, and red denotes high seroreactivity to probed fragments. Lacunae present across more than one comparison are designated by a triangle.

Five lacunae were identified across more than one SMA comparison, including one CD36-binding extracellular PfEMP1 fragment (PFD0625c, a Group C PfEMP1), one non-CD36-binding extracellular PfEMP1 fragment (the *var1*-encoded PFE1640w), an MSP1 fragment, and two AMA1s that were different concentrations of a 3D7-based vaccine preparation (Fig. 6). No fragments were identified as lacunae across all three acute SMA sera comparisons, but the latter four fragments were lacunae in acute SMA comparisons with convalescent SMA sera and with uncomplicated malaria control sera.

#### Cerebral malaria and severe malarial anaemia

**Figure 7:**
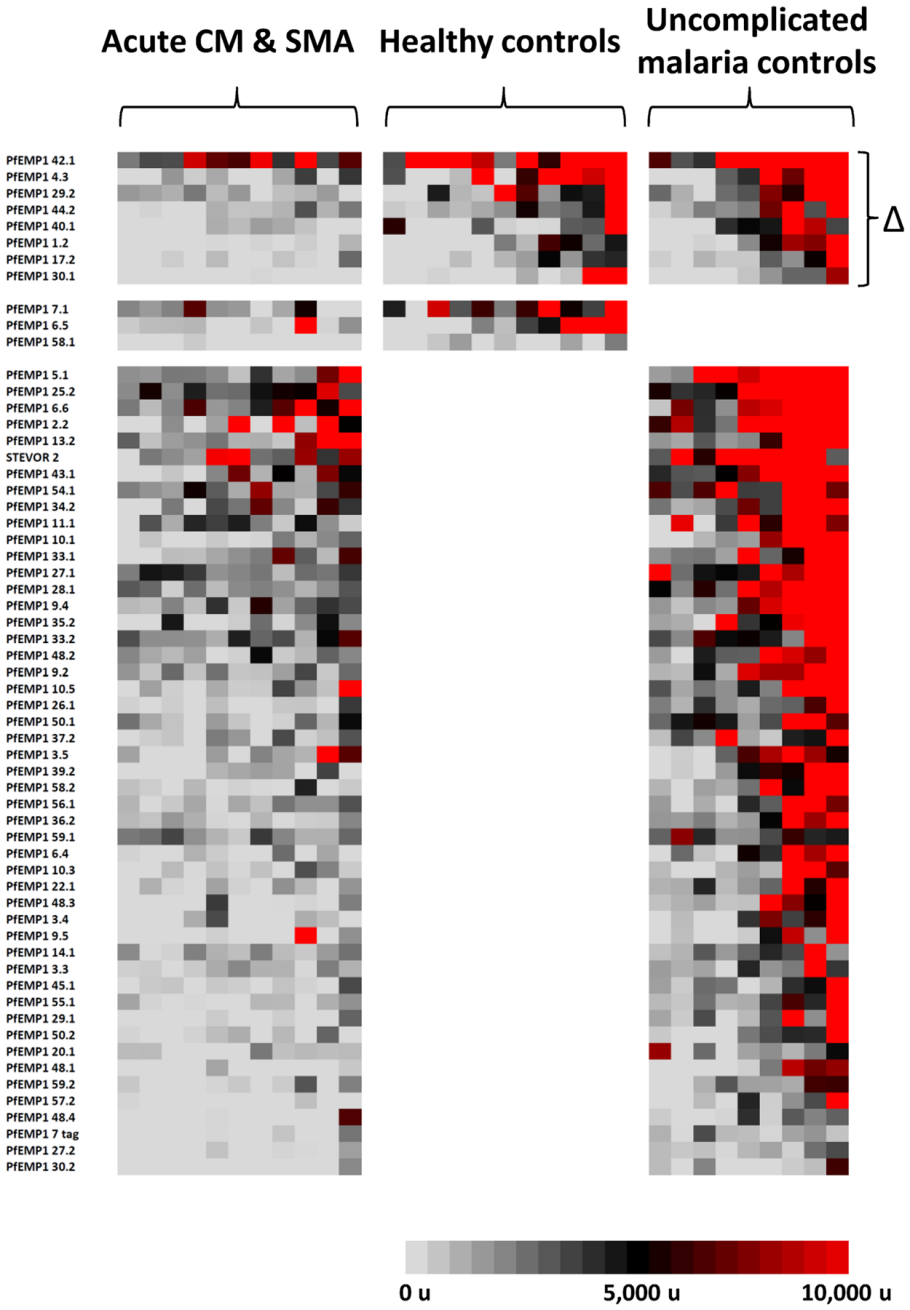
Heat map of lacunae shared across different acute sera comparisons of CM + SMA subjects. *P. falciparum* antigen lacunae were grouped by recurrence across different comparisons with acute sera from subjects with cerebral malaria and severe malarial anaemia. Each column displays the seroreactivity profile of one serum sample, and each row displays the seroreactivity profile of an individual protein fragment. Grey color indicates no seroreactivity, black is low-to-moderate seroreactivity, and red denotes high seroreactivity to probed fragments. Lacunae present across more than one comparison are designated by a triangle.

Eight lacunae were identified across more than one CM + SMA comparison, all of which were extracellular PfEMP1 fragments, including two non-CD36-binding fragments (Fig. 7): a DBLβ-DBLγ domain couplet from MAL6P1.316 associated with DC8 and the proximal portion of PF11_0008, a DC5 PfEMP1.

### Area under the curve analyses

Seroreactivity to no single malaria protein fragment reliably distinguished cerebral malaria cases from uncomplicated malaria controls or distinguished severe malarial anaemia cases from uncomplicated malaria controls (Table S4). However, seroreactivity to several different malaria protein fragments distinguished children with both cerebral malaria and severe malarial anaemia from uncomplicated malaria controls (Table S4). Four fragments had receiver operating characteristic curves with areas under the curve of at least 0.900; all of these fragments were from extracellular PfEMP1s. The capacity to distinguish severe malaria cases from controls did not consistently improve using seroreactivity to subsets of malaria protein fragments, regardless of the number of fragments, for any type of severe malaria (Table S5).

## Discussion

Identifying specific gaps in immunity to parasite variant surface antigens (VSA) in children with severe malaria has the potential to pinpoint antigens critical to cerebral malaria and severe malarial anaemia pathogenesis. Antigens targeted by protective natural immunity may be promising targets for a vaccine to prevent severe malaria in children. Using a custom protein microarray to measure seroreactivity to fragments of the major *P. falciparum* VSA PfEMP1s, we found that sera from Malian children with severe malaria recognized fewer extracellular PfEMP1 fragments and reacted to particular fragments less intensely than control sera. Sera from children with severe malaria also reacted less to several members of the RIFIN and STEVOR VSA families. No such gaps (“lacunae”) in seroreactivity were consistently identified for several malaria blood stage vaccine candidate antigens, supporting the central role of VSAs in immunity against severe malaria. Children with severe malarial anaemia had fewer VSA fragment lacunae than did children with cerebral malaria. Children who had both cerebral malaria and severe malarial anaemia shared some lacunae with children who had only cerebral malaria and children who had only severe malarial anaemia, suggesting that there are both syndrome-specific and non-specific targets of protective immunity.

Convalescent sera following recovery from cerebral malaria or severe malarial anaemia reacted more intensely than acute sera to certain non-CD36-binding PfEMP1s; again, this was not the case for other malaria antigens, potentially implicating this subset of VSAs in the pathogenesis of severe disease. All such fragments for cerebral malaria cases were from CIDRα1-containing PfEMP1s; three out of four PfEMP1s in severe malarial anaemia cases were from CIDRα1-containing PfEMP1s. However, only one differentially seroreactive fragment contained the actual CIDRα1 domain; the rest contained consecutive pairs of DBL domains distal to the CIDRα1 domain.

One potential explanation for the failure to react more intensely to a domain that may be critical to severe malaria pathogenesis is that a single episode of severe malaria may be insufficient to develop protective immunity to severe malaria in all individuals. While mathematical modelling has indicated that immunity to severe malaria may be acquired after one or two infections^30^, recent studies support the idea that acquisition of immunity to severe malaria may be more gradual^33^. Indeed, studies have identified subsets of children who experienced multiple episodes of severe malaria^34,35^. An alternative explanation is that the specific PfEMP1 domains that are exposed to the immune system and generate antibody responses that are temporally associated with the acquisition of natural immunity are not the PfEMP1 domains that are critical to receptor binding and severe malaria pathogenesis. This may be the case with VAR2CSA domains associated with immunity to pregnancy-associated malaria, which appear to differ from those VAR2CSA domains critical to binding placental receptors^36^. In such a scenario, CIDRα1 domains associated with EPCR binding would not be associated with immune system exposure and a subsequent increase in seroreactivity, while other constitutive domains on the same PfEMP1 would be exposed to and elicit a response by the immune system. This would explain why fragments identified in the acute-convalescent comparison were primarily DBL-DBL domain couplets distal to CIDRα1 domains. Alternatively, the CIDRα1 domain may be exposed to the immune system, but somehow fails to elicit an appropriate response, in contrast to neighboring constitutive domains.

One potential explanation for why children who served as controls had uncomplicated malaria is that they had immunity against severe malaria, so that infection produced only relatively mild symptoms. If this were the case, comparisons of sera from cases and controls may have identified gaps in immunologic protection in children with severe malaria. Lacunae that were “filled in” after an episode of either cerebral malaria or severe malarial anaemia consisted of a few extracellular PfEMP1 fragments, while many additional immunity gaps remained in comparison to uncomplicated malaria controls. Whether this post-episode immunity is sufficient to protect these children against subsequent severe malaria episodes is unclear. Consistent with the models predicting a more gradual acquisition of protective immunity to severe malaria^33^, we found that serological profiles of children who had recovered from severe malaria still did not resemble those of their matched peers who experienced episodes of uncomplicated malaria. Other potential explanations for why a child does not develop severe malaria, such as earlier treatment, may also explain differences in severity of disease.

Comparisons with healthy controls may be harder to interpret than comparisons with uncomplicated malaria controls, given that the immune status of healthy controls is unknown. Healthy controls could have protective immunity against severe malaria, or they may simply be unexposed to infection. In comparison to healthy controls, children with cerebral malaria had multiple PfEMP1 lacunae, as did, to a lesser extent, children with both cerebral malaria and severe malarial anaemia. In contrast, children with severe malarial anaemia had only two lacunae compared to healthy controls, making it appear that these children differed from healthy controls to a lesser extent than children with cerebral malaria. Alternatively, decreased exposure and thus decreased acquisition of immunity in the healthy control group could explain this observation, with “healthy” unexposed children and those with anaemia being more similar because both have less immunity. Indeed, healthy controls recognized fewer merozoite proteins than severe malarial anaemia cases, suggesting a low level of malaria exposure in this group, in contrast to the healthy controls for cerebral malaria subjects.

Lacunae that recurred across different analyses were predominantly seen with respect to extracellular PfEMP1s, suggesting that these antibody responses play a critical role in defining vulnerability to severe malaria; intracellular PfEMP1s were identified as lacunae solely in comparisons of cerebral malaria and uncomplicated malaria in sera collected at the acute visit. We have previously shown that intracellular PfEMP1 seroreactivity increases with age and over the course of a malaria season^37^. This difference in reactivity to intracellular PfEMP1s suggests a greater degree of malaria exposure in controls with uncomplicated malaria versus matched cerebral malaria cases.

Previous work has suggested expression of distinct subsets of non-CD36-binding PfEMP1s in cerebral malaria and severe malarial anaemia cases^21,29^. In this study, following an episode of cerebral malaria or severe malarial anaemia, children “filled in” several PfEMP1 lacunae, but the majority of lacunae that separated them from uncomplicated malaria controls remained unfilled. An ordered pattern of acquisition of immunity to particular PfEMP1 domains was seen in young Tanzanian children as they aged^38,39^, with early acquisition of immunity to non-CD36-binding PfEMP1 domains. We found that differences between sera from acute cerebral malaria and severe malarial anaemia cases and their convalescent sera were primarily due to non-CD36-binding PfEMP1s, particularly non-CD36-binding PfEMP1s with CIDRα1 domains. However, acute sera from severe malaria cases also differed with controls with respect to CD36-binding PfEMP1 fragments. The extent to which seroreactivity to CD36-binding PfEMP1 fragments is important in protection against severe malaria is unclear and requires further investigation.

Next steps to identify lacunae critical to protection against severe malaria include identifying the PfEMP1 variants expressed in children with severe malaria, determining their complete sequences, and characterizing which PfEMP1 domains are critical to protection against such infections. An approach combining next generation sequencing to identify these PfEMP1s and then populating a protein microarray with protein fragments of their constitutive domains will provide a powerful tool to assay the immunity of children with severe malaria during an acute attack and determine which lacunae are subsequently filled. With this approach, we may better identify particular VSA fragments with differential seroreactivity that may allow us to identify children vulnerable to cerebral malaria or severe malarial anaemia.

This study had some limitations. A protein microarray was populated with PfEMP1 fragments from the reference strain 3D7, representing only a subset of the known DBL and CIDR domain types, as well as domain cassettes^31^. In particular, DC8, DC13, and DC17 have been associated with severe malaria, but are either not found or are incomplete in the 3D7 *var* gene repertoire. Future protein microarray studies of severe malaria should include representative fragments from these and other DCs underrepresented in 3D7.

Protein fragments on our microarray had an upper size limit of about 1000 amino acids. Domain-domain interactions that do not fit within these constraints would not be identified with this approach. In addition, the fidelity of protein conformation could be an issue when using an *E. coli*-based cell-free system that allows formation of disulphide bonds. Using this system, we have observed epidemiological patterns matching what would be predicted in the acquisition of natural immunity, including patterns of recognition of VAR2CSA, the leading PfEMP1 candidate for a malaria vaccine, in malaria-exposed Malian women of differing gravidities^36^. Nevertheless, protein conformations may not completely match native PfEMP1 conformations.

Development of an effective severe malaria vaccine relies on identification of malaria proteins critical to the acquisition of immunity to specific severe malaria syndromes. Identification of these proteins requires a broad approach examining multiple antigens at once, a strength of microarray-based methods. This approach may allow further winnowing of the thousands of known variant surface antigens to determine a much smaller subset eliciting increased seroreactivity by children protected against severe malaria that may form the basis of a vaccine or therapy. This could also allow for development of an antibody test to identify those children most susceptible to severe malaria in a population.

## Methods

### Study Setting

The study was conducted in Bandiagara, a town of 13,634 inhabitants (2002 census) in the Dogon Country in east-central Mali. Bandiagara is relatively dry, with a mean annual rainfall of 600 mm. *Anopheles gambiae* is the principal malaria vector. Malaria transmission is highly seasonal, with minimal transmission at the height of the dry season in March; less than one infected mosquito bite per person per month as the transmission season starts and ends in June and December, respectively; and a peak of up to 60 infected bites per person per month^3,40^. *P. falciparum* represents 97% of malaria infections with 3% due to *P. malariae* and rare infections with *P. ovale*. Despite a seasonal transmission pattern, the malaria burden is heavy: children aged less than 10 years had an average of two clinical malaria episodes in the 1999 transmission season^40^, and severe malaria afflicts 1 in 50 children aged less than 6 years each year^3^. Older children and adults are relatively protected against malaria disease, but remain susceptible to infection.

### Ethics Statement

The protocol was approved by institutional review boards of the Faculty of Medicine, Pharmacy and Dentistry, Bamako, Mali and the University of Maryland, Baltimore. Written informed consent was obtained from parents or guardians of all study participants. All methods were performed in accordance with the relevant guidelines and regulations.

### Participants

Cases of severe malaria from Bandiagara and surrounding areas were admitted to the Bandiagara Malaria Project ward from October 1999 to December 2002. Cases were classified as severe malaria based on modified criteria put forth by the World Health Organization^41^. An episode of cerebral malaria was defined using the standard WHO definition of cerebral malaria: a Blantyre Coma Score of <3^42^, with no other obvious cause of coma^43^. Severe malarial anaemia was defined as a haemoglobin ≤ 5 g/dL. An additional subset of severe malaria cases was defined as cases with characteristics of both cerebral malaria and severe malarial anaemia. No subjects received blood transfusions as part of their care. As previously described, each index case was age-, residence-, and ethnicity-matched to a case of uncomplicated malaria and a healthy control^44^. Age categories were defined as 3–5 months, 6–11 months, 1 year, 2 years, 3–4 years, 5–6 years, 7–8 years, 9–10 years, 11–12 years, and 13–14 years. Residence was defined as one of eight distinct sectors of Bandiagara town or, in the case of children from outer villages, the specific village of origin. Uncomplicated malaria was defined as *P. falciparum* parasitemia and an axillary temperature of 37.5 °C detected by active surveillance, or parasitemia and symptoms leading to treatment-seeking behavior in the absence of other clear cause of fever on passive surveillance.

Matched uncomplicated malaria controls were enrolled from the population of children presenting to the daily Bandiagara Malaria Project clinic. Healthy controls were enrolled by traveling to the home of a child with severe malaria. We then followed a standardized routine of exiting the front entrance of the child’s housing compound and making consecutive left turns until another compound with an eligible control was identified. Children were enrolled as healthy controls if they were asymptomatic for acute illness, had no evidence or history of chronic illness, and if they were found to be aparasitemic upon examination.

Sera was obtained at enrolment (June to December) and, when possible, in the following dry season, a period extending from January to June (“convalescent sera”), typically several months after the acute illness.

### Protein microarray

A protein microarray was made with 170 fragments of PfEMP1s, six full-length STEVOR antigens, and three full-length RIFIN antigens, all based on the 3D7 reference genome (data file S1). For comparison, 268 diverse apical membrane 1 (AMA1) fragments, 22 merozoite surface protein 1 (MSP1) fragments, and 30 RH5 fragments were also included on the array, based on sequences derived from field samples. Eighty percent of all extracellular 3D7 PfEMP1 domains were successfully cloned and printed, typically as fragments comprised of paired consecutive domains (Diagram S1). The 170 PfEMP1 fragments included fragments from all 13 3D7 non-CD36-binding PfEMP1s and from 44 of the 46 CD36-binding 3D7 PfEMP1s.

#### Microarray construction and controls

Protein microarray construction followed a four-step process that includes: (1) PCR amplification of each complete or partial *P. falciparum* open reading frame, (2) *in vivo* recombination cloning, (3) *in vitro* transcription/translation, and (4) microarray chip printing^45^.

### Statistical analysis

Fluorescence intensity was defined as the raw signal intensity reduced by the mean for the no-DNA negative controls. We used data analysis techniques that have previously been used for evaluating humoral immune responses in *P. falciparum* protein microarrays^7^. “Recognition” of a protein fragment was defined as fluorescence intensity significantly greater than that of the malaria-naïve control group, based on a two-sample Kolmogorov-Smirnov test, as per previous array analyses^36^. Comparisons of recognized proteins between severe malaria cases and controls in Fig. 1 were performed by a McNemar’s test.

Fluorescence intensity for each *P. falciparum* antigen variant was compared for severe malaria cases versus controls to identify protein fragments eliciting more reactivity by sera from control groups versus severe malaria cases, thereby defining “lacunae” in antibody responses of children with severe malaria. The significance of these comparisons was determined by a Wilcoxon-signed rank test. Similarly, fluorescence intensity for each *P. falciparum* antigen variant was compared for severe malaria at the time of illness versus convalescence to identify “lacunae” in antibody responses when presenting with severe malaria.

All P-values presented were two-sided. In identifying lacunae, consistent with other similar published analyses, we did not adjust P-values for multiple comparisons, instead reporting all results with a P < 0.05 and noting comparisons with a P < 0.01 or P < 0.001, as in other microarray analyses^36,37,46^. Statistical analyses were primarily conducted using SAS 9.2 and the Real Statistics Resource Pack software (Release 3.8).

To identify individual protein fragments that elicited seroreactivities predictive of whether an individual had severe malaria versus uncomplicated malaria, we employed a leave one out cross-validation (LOOCV) approach, generating receiver operating characteristic curve plots for each fragment and comparing results by the area-under-the-curve^47^. This analysis was performed for individual protein fragments (Table S4) and for increasing feature counts up to ten fragments (Table S5).

### Data availability

The microarray dataset of fluorescence intensities to parasite protein fragments generated during during the current study are attached as a supplementary data file (Database S1).

## Electronic supplementary material

Supplementary information

Data file S1

Database S1

## References

[1] Murphy SC, Breman JG (2001). Gaps in the childhood malaria burden in Africa: cerebral malaria, neurological sequelae, anemia, respiratory distress, hypoglycemia, and complications of pregnancy. Am J Trop Med Hyg.

[2] Snow RW, Marsh K (2002). The consequences of reducing transmission of Plasmodium falciparum in Africa. Adv. Parasitol.

[3] Lyke KE (2004). Incidence of severe Plasmodium falciparum malaria as a primary endpoint for vaccine efficacy trials in Bandiagara, Mali. Vaccine.

[4] Djimde A (2001). A molecular marker for chloroquine-resistant falciparum malaria. N. Engl. J. Med.

[5] Chan JA, Fowkes FJ, Beeson JG (2014). Surface antigens of Plasmodium falciparum-infected erythrocytes as immune targets and malaria vaccine candidates. Cell Mol Life Sci.

[6] Chan JA (2012). Targets of antibodies against Plasmodium falciparum-infected erythrocytes in malaria immunity. J Clin Invest.

[7] Crompton PD (2010). A prospective analysis of the Ab response to Plasmodium falciparum before and after a malaria season by protein microarray. Proc. Natl. Acad. Sci. USA.

[8] Marsh K, Otoo L, Hayes RJ, Carson DC, Greenwood BM (1989). Antibodies to blood stage antigens of Plasmodium falciparum in rural Gambians and their relation to protection against infection. Trans. R. Soc. Trop. Med. Hyg.

[9] Oh SS (2000). Plasmodium falciparum erythrocyte membrane protein 1 is anchored to the actin-spectrin junction and knob-associated histidine-rich protein in the erythrocyte skeleton. Mol. Biochem. Parasitol.

[10] Baruch DI (1995). Cloning the P. falciparum gene encoding PfEMP1, a malarial variant antigen and adherence receptor on the surface of parasitized human erythrocytes. Cell.

[11] Gardner MJ (2002). Genome sequence of the human malaria parasite Plasmodium falciparum. Nature.

[12] Newbold CI (1997). PfEMP1, polymorphism and pathogenesis. Ann. Trop. Med. Parasitol.

[13] Rogerson SJ, Chaiyaroj SC, Ng K, Reeder JC, Brown GV (1995). Chondroitin sulfate A is a cell surface receptor for Plasmodium falciparum-infected erythrocytes. J. Exp. Med.

[14] Smith JD (1995). Switches in expression of Plasmodium falciparum var genes correlate with changes in antigenic and cytoadherent phenotypes of infected erythrocytes. Cell.

[15] Su XZ (1995). The large diverse gene family var encodes proteins involved in cytoadherence and antigenic variation of Plasmodium falciparum-infected erythrocytes. Cell.

[16] Treutiger CJ, Heddini A, Fernandez V, Muller WA, Wahlgren M (1997). PECAM-1/CD31, an endothelial receptor for binding Plasmodium falciparum-infected erythrocytes. Nat. Med.

[17] Kyriacou HM (2006). Differential var gene transcription in Plasmodium falciparum isolates from patients with cerebral malaria compared to hyperparasitaemia. Mol. Biochem. Parasitol.

[18] Avril M (2012). A restricted subset of var genes mediates adherence of Plasmodium falciparum-infected erythrocytes to brain endothelial cells. Proc. Natl. Acad. Sci. USA.

[19] Bengtsson A (2013). A novel domain cassette identifies Plasmodium falciparum PfEMP1 proteins binding ICAM-1 and is a target of cross-reactive, adhesion-inhibitory antibodies. J Immunol.

[20] Claessens A (2012). A subset of group A-like var genes encodes the malaria parasite ligands for binding to human brain endothelial cells. Proc. Natl. Acad. Sci. USA.

[21] Lavstsen T (2012). Plasmodium falciparum erythrocyte membrane protein 1 domain cassettes 8 and 13 are associated with severe malaria in children. Proc Natl Acad Sci USA.

[22] Lennartz F (2017). Structure-Guided Identification of a Family of Dual Receptor-Binding PfEMP1 that Is Associated with Cerebral Malaria. Cell Host Microbe.

[23] Turner L (2013). Severe malaria is associated with parasite binding to endothelial protein C receptor. Nature.

[24] Fried M, Duffy PE (1996). Adherence of Plasmodium falciparum to chondroitin sulfate A in the human placenta. Science.

[25] Salanti A (2004). Evidence for the involvement of VAR2CSA in pregnancy-associated malaria. J Exp Med.

[26] Salanti A (2003). Selective upregulation of a single distinctly structured var gene in chondroitin sulphate A-adhering Plasmodium falciparum involved in pregnancy-associated malaria. Mol Microbiol.

[27] Wang CW (2012). Evidence for *in vitro* and *in vivo* expression of the conserved VAR3 (type 3) plasmodium falciparum erythrocyte membrane protein 1. Malar J.

[28] Zhang Y (2014). The var3 genes of Plasmodium falciparum 3D7 strain are differentially expressed in infected erythrocytes. Parasite.

[29] Warimwe GM (2009). Plasmodium falciparum var gene expression is modified by host immunity. Proc. Natl. Acad. Sci. USA.

[30] Gupta S, Snow RW, Donnelly CA, Marsh K, Newbold C (1999). Immunity to non-cerebral severe malaria is acquired after one or two infections. Nat. Med.

[31] Rask, T. S., Hansen, D. A., Theander, T. G., Gorm Pedersen, A. & Lavstsen, T. Plasmodium falciparum erythrocyte membrane protein 1 diversity in seven genomes–divide and conquer. *PLoS Comput Biol* **6**, 10.1371/journal.pcbi.1000933 (2010). 10.1371/journal.pcbi.1000933 PMC2940729 20862303

[32] Bertin GI (2013). Expression of the domain cassette 8 Plasmodium falciparum erythrocyte membrane protein 1 is associated with cerebral malaria in Benin. PLoS. ONE.

[33] Griffin JT (2015). Gradual acquisition of immunity to severe malaria with increasing exposure. Proc. Biol. Sci.

[34] Goncalves BP (2014). Parasite burden and severity of malaria in Tanzanian children. N. Engl. J Med.

[35] Mwangi TW (2008). Evidence for over-dispersion in the distribution of clinical malaria episodes in children. PLoS. ONE.

[36] Travassos MA (2015). Differential Recognition of Terminal Extracellular Plasmodium falciparum VAR2CSA Domains by Sera from Multigravid, Malaria-Exposed Malian Women. Am J Trop Med Hyg.

[37] Travassos MA (2013). Seroreactivity to Plasmodium falciparum erythrocyte membrane protein 1 intracellular domain in malaria-exposed children and adults. J. Infect. Dis.

[38] Cham GK (2009). Sequential, ordered acquisition of antibodies to Plasmodium falciparum erythrocyte membrane protein 1 domains. J. Immunol.

[39] Cham, G. K. *et al*. Hierarchical, domain type-specific acquisition of antibodies to Plasmodium falciparum erythrocyte membrane protein 1 in Tanzanian children. *Infect. Immun* (2010). 10.1128/IAI.00593-10 PMC2976311 20823214

[40] Coulibaly D (2002). Impact of preseason treatment on incidence of falciparum malaria and parasite density at a site for testing malaria vaccines in Bandiagara, Mali. Am. J. Trop. Med. Hyg.

[41] Severe falciparum malaria (2000). World Health Organization, Communicable Diseases Cluster. Trans. R. Soc. Trop Med Hyg.

[42] WHO. *Guidelines for the treatment of malaria*. (World Health Organization, 2010). 25473692

[43] Taylor TE (2009). Caring for children with cerebral malaria: insights gleaned from 20 years on a research ward in Malawi. Trans. R. Soc. Trop. Med. Hyg.

[44] Lyke KE (2003). Association of intraleukocytic Plasmodium falciparum malaria pigment with disease severity, clinical manifestations, and prognosis in severe malaria. Am J Trop Med Hyg.

[45] Davies DH (2005). Profiling the humoral immune response to infection by using proteome microarrays: high-throughput vaccine and diagnostic antigen discovery. Proc. Natl. Acad. Sci. USA.

[46] Bailey, J. A. *et al*. Seroreactivity to a large panel of field-derived *Plasmodium falciparum* blood stage antigen variants reflects seasonal and lifetime acquired immunity to malaria. *Am J Trop Med Hyg* (2014). 10.4269/ajtmh.14-0140 PMC4347399 25294612

[47] Lessa-Aquino C (2013). Identification of seroreactive proteins of Leptospira interrogans serovar copenhageni using a high-density protein microarray approach. PLoS Negl Trop Dis.

