# Supplementary material for: Children with cerebral malaria or severe malarial anaemia lack immunity to distinct variant surface antigen subsets
Source: Sci Rep. 2018 Apr 19;8:6281. doi: 10.1038/s41598-018-24462-4 (PMC5908851; doi:10.1038/s41598-018-24462-4)
Supplement: Supplementary file 1 — Supplementary information [file 41598_2018_24462_MOESM1_ESM.pdf]

Children with cerebral malaria or severe malarial anaemia lack immunity to distinct variant surface antigen subsets

Authors: Mark A. Travassos[1*], Amadou Niangaly[2], Jason A. Bailey[1], Amed Ouattara[1], Drissa Coulibaly[2], Kirsten E. Lyke[1], Matthew B. Laurens[1], Jozelyn Pablo[3], Algis Jasinskas[3], Rie Nakajima[3], Andrea A. Berry[1], Matthew Adams[1], Christopher G. Jacob[4], Andrew Pike[1], Shannon Takala-Harrison[1], Li Liang[3], Bourema Kouriba[2], Abdoulaye K Kone[2], J. Alexandra Rowe[5], JoAnn Moulds[6], Dapa A. Diallo[2], Ogobara K. Doumbo[2], Mahamadou A. Thera[2], Philip L. Felgner[3], and Christopher V. Plowe[1]

Supplementary Figures

Fig S1. Acute and convalescent recognition of parasite antigens for children with (A) CM and (B) SMA. * indicates a significant difference in percentage of recognized fragments between acute and convalescent sera ($P < 0.05$, McNemar's test).

Fig S2. Recognition of *P. falciparum* antigens for subjects with CM versus SMA versus CM + SMA. * indicates a significant difference in percentage of recognized fragments with subjects with cerebral malaria ($P < 0.05$, McNemar's test). Sera from subjects with severe malarial anaemia did not differ with sera from subjects with both cerebral malaria and severe malarial anaemia in breadth of recognition comparisons.

Fig S3. Differentially seroreactive *P. falciparum* antigens for sera from uncomplicated malaria controls versus severe malaria subjects. Sera from uncomplicated malaria controls reacted more intensely to 107, 19, and 57 fragments versus sera from subjects with cerebral malaria, severe malarial anaemia, and both cerebral malaria and severe

malarial anaemia, respectively. Non-CD36-binding PfEMP1 fragments are designated with an "S". * indicates P $\leq$ 0.05; ** P < 0.01, and *** P < 0.001 (Wilcoxon's signed-rank test).

Supplementary Tables

Table S1. Differentially seroreactive *P. falciparum* fragments in comparisons between uncomplicated malaria controls and severe malaria cases.

Table S2. Differentially seroreactive *P. falciparum* fragments in comparisons between healthy controls and severe malaria cases.

Table S3. Differentially seroreactive *P. falciparum* fragments in comparisons between acute and convalescent severe malaria cases.

Table S4. Area under the curve for the top antigens distinguishing severe malaria groups from uncomplicated malaria controls. The ten antigens with the highest AUCs for the standard and inverse analyses are listed, as well as any additional antigens with an AUC > 0.700.

Table S5. Area under the curve for each feature count for leave one out cross-validation (LOOCV) analyses of antigens distinguishing severe malaria cases from uncomplicated malaria controls.

Diagram S1. Each microarray PfEMP1 fragment mapped within the 3D7 PfEMP1 repertoire. Location is given by amino acid coordinates. CIDR = Cysteine interdomain

rich region domain; DBL = Duffy-binding like (DBL) domain; ATS = acidic terminal segment

Data file S1. List of microarray protein fragments.

Database S1. Microarray fluorescence intensities to parasite protein fragments.

Figure S1.

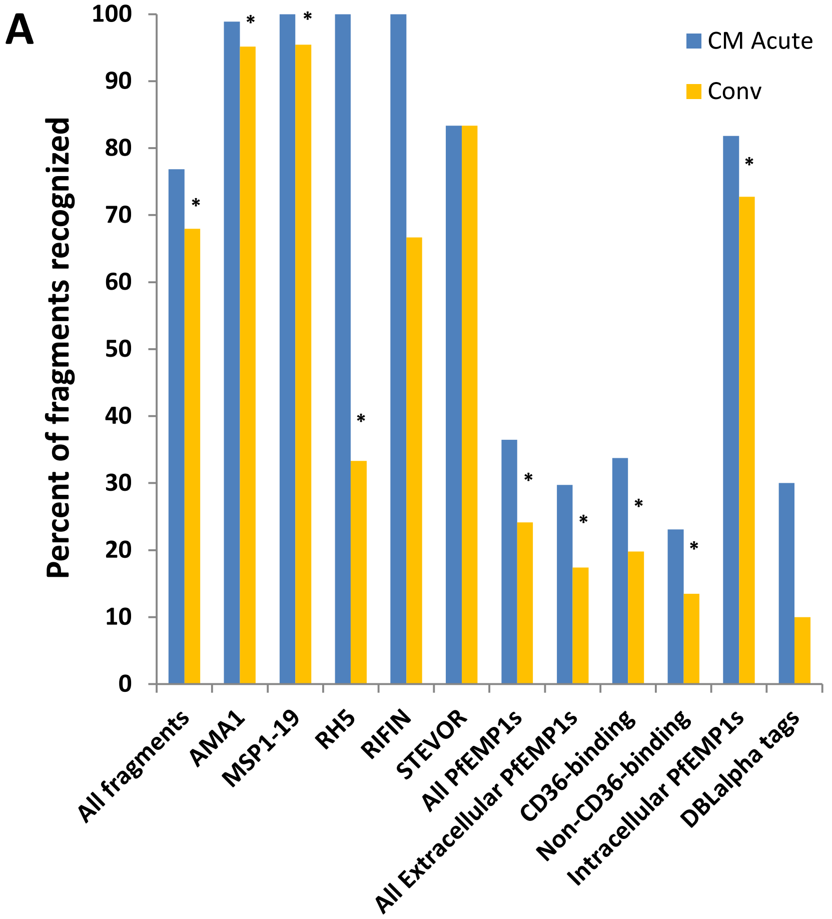
A
Percent of fragments recognized
100
90
80
70
60
50
40
30
20
10
0
CM Acute
Conv
All fragments
AMA1
MSP1-19
RH5
RIFIN
STEVOR
All PfEMP1s
All Extracellular PfEMP1s
CD36-binding
Non-CD36-binding
Intracellular PfEMP1s
DBLalpha tags

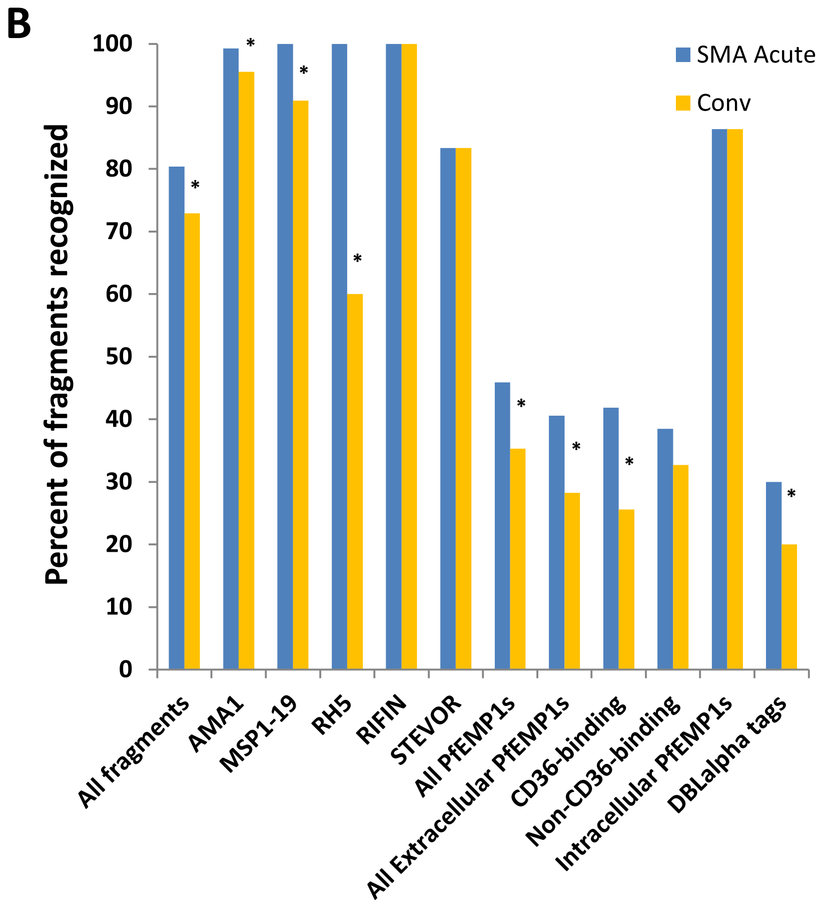
B
Percent of fragments recognized
100
90
80
70
60
50
40
30
20
10
0
SMA Acute
Conv
All fragments
AMA1
MSP1-19
RH5
RIFIN
STEVOR
All PfEMP1s
All Extracellular PfEMP1s
CD36-binding
Non-CD36-binding
Intracellular PfEMP1s
DBLalpha tags

Figure S2.

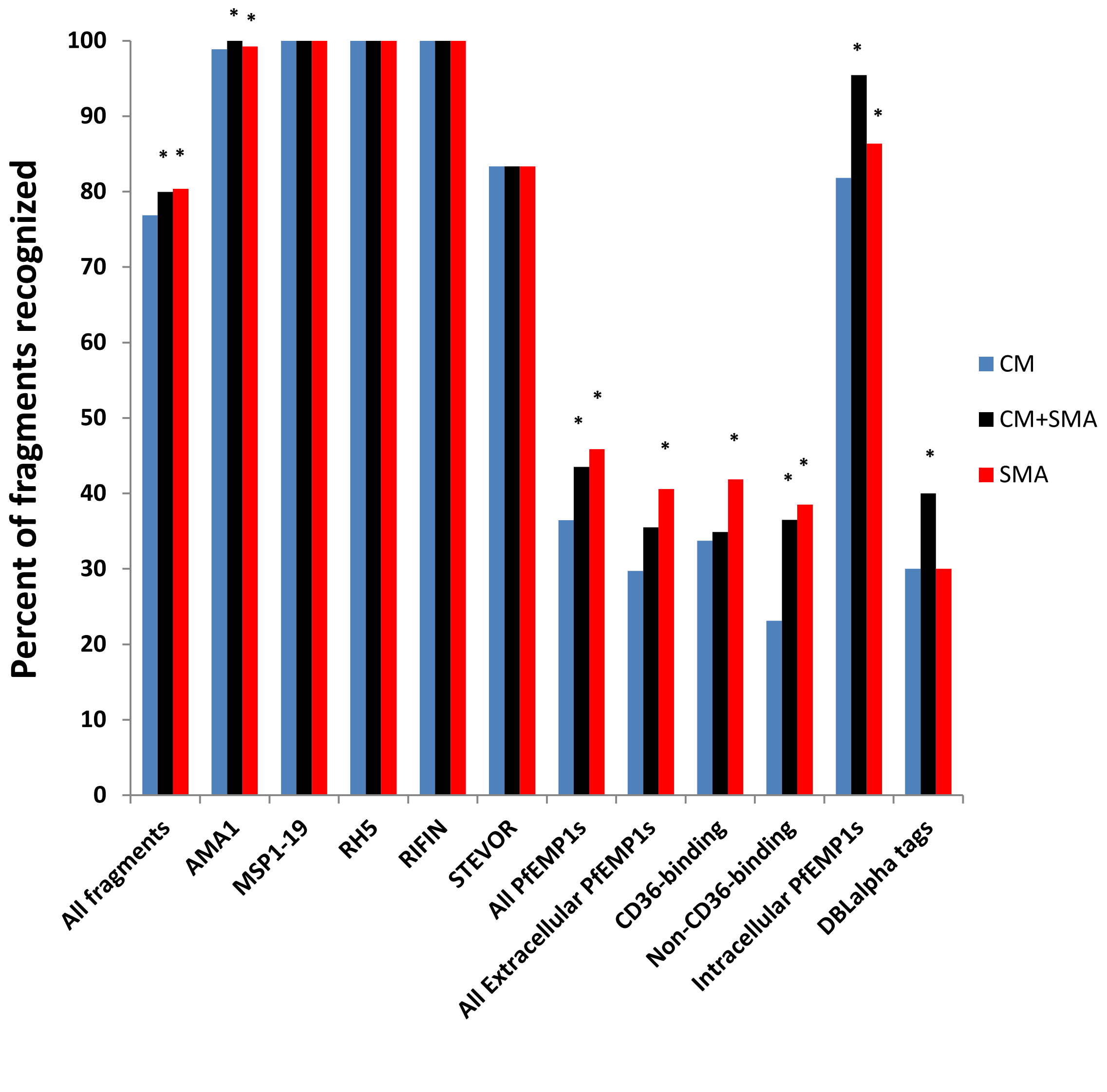
Percent of fragments recognized
100
90
80
70
60
50
40
30
20
10
0
All fragments
AMA1
MSP1-19
RH5
RIFIN
STEVOR
All PfEMP1s
All Extracellular PfEMP1s
CD36-binding
Non-CD36-binding
Intracellular PfEMP1s
DBLalpha tags
CM
CM+SMA
SMA

Figure S3.

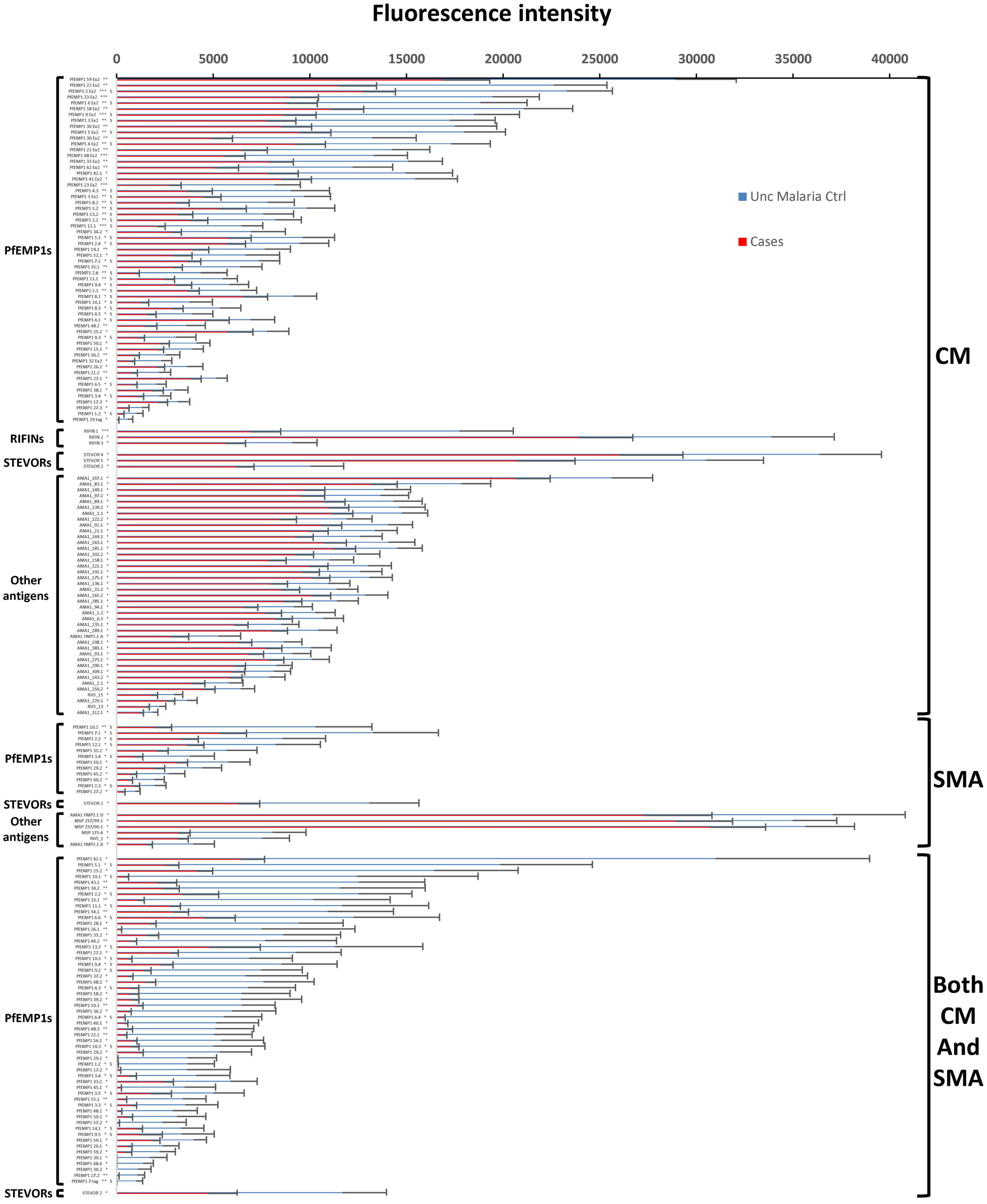

Fluorescence intensity
0
5000
10000
15000
20000
25000
30000
35000
40000
Unc Malaria Ctrl
Cases
PfEMP1s
RIFINs
STEVORs
Other antigens
CM
PfEMP1s
STEVORs
Other antigens
SMA
PfEMP1s
STEVORs
Both CM And SMA

Table S1. Differentially seroreactive *P. falciparum* fragments in comparisons between uncomplicated malaria controls and severe malaria cases.

	Total fragments	Cerebral malaria	Severe malarial anemia	CM+SMA	Shared fragments: CM & SMA	Shared fragments: CM & CM+SMA	Shared fragments: SMA & CM+SMA	Shared fragments across all 3 comparisons
PfEMP1	170	34.7%	7.1%	32.9%	2.9%	9.4%	3.5%	1.8%
STEVOR	6	50.0%	16.7%	16.7%	16.7%	16.7%	16.7%	16.7%
RIFIN	3	100%	0	0	0	0	0	0
AMA1	268	14.6%	0.7%	0	0.4%	0	0	0
MSP1-19	22	0	13.6%	0	0	0	0	0
RH5	15	13.3%	6.7%	0	0	0	0	0

Table S2. Differentially seroreactive *P. falciparum* fragments in comparisons between healthy controls and severe malaria cases.

	Total fragments	Cerebral malaria	Severe malarial anemia	CM+SMA	Shared fragments: CM & SMA	Shared fragments: CM & CM+SMA	Shared fragments: SMA & CM+SMA	Shared fragments across all 3 comparisons
PfEMP1	170	14.7%	1.2%	6.5%	0	2.9%	0	0
STEVOR	6	0	0	0	0	0	0	0
RIFIN	3	0	0	0	0	0	0	0
AMA1	268	0.4%	0	0	0	0	0	0
MSP1-19	22	0	0	0	0	0	0	0
RH5	15	0	0	0	0	0	0	0

Table S3. Differentially seroreactive *P. falciparum* fragments in comparisons between acute and convalescent severe malaria cases.

	Total fragments	Cerebral malaria	Severe malarial anemia	Shared fragments: CM & SMA
PfEMP1	170	2.4%	2.4%	0.6%
STEVOR	6	0	0	0
RIFIN	3	0	0	0
AMA1	268	0	0.7%	0
MSP1-19	22	0	9.1%	0
RH5	15	0	0	0

Table S4. Area under the curve for the top antigens distinguishing severe malaria groups from uncomplicated malaria controls. The ten antigens with the highest AUCs for the standard and inverse analyses are listed, as well as any additional antigens with an AUC ≥ 0.700.

Standardized Antigen name	Antigen	Group	AUC	P-value
		**Cerebral malaria**		
MSP 189-6	MSP 189-6	MSP1-19	0.633	0.034
PfEMP1 29 tag	PF08_0106	DBLalpha tags	0.394	0.007
PfEMP1 1.2	MAL6P1316_2	PfEMP1 - Extracellular - non-CD36-binding	0.380	0.026
PfEMP1 10.3	PFE1640w_4	PfEMP1 - Extracellular - non-CD36-binding	0.380	0.048
AMA1_221.1	AMA1_221.1	AMA1	0.377	0.050
AMA1_105.2	AMA1_105.2	AMA1	0.377	0.050
PfEMP1 4.4	PF11_0008e1s2	PfEMP1 - Extracellular - non-CD36-binding	0.376	0.040
PfEMP1 49.2	PFD1005c_2	PfEMP1 - Extracellular - CD36-binding	0.376	0.047
AMA1_137.2	AMA1_137.2	AMA1	0.375	0.046
PfEMP1 36.1	PF13_0364_1	PfEMP1 - Extracellular - CD36-binding	0.375	0.047
PfEMP1 21.2	MAL7P155_2	PfEMP1 - Extracellular - CD36-binding	0.721 (inv)	< 0.001
PfEMP1 12.1	PFI1820w_1	PfEMP1 - Extracellular - non-CD36-binding	0.719 (inv)	< 0.001
RIFIN 1	MAL7P1.215	RIFIN	0.704 (inv)	0.001
PfEMP1 48.2	PFD0995c_2	PfEMP1 - Extracellular - CD36-binding	0.700 (inv)	0.001
PfEMP1 2 Ex2	PF08_0140e2s1	PfEMP1 - Intracellular	0.698 (inv)	0.002
PfEMP1 5.2	PF11_0521_3	PfEMP1 - Extracellular - non-CD36-binding	0.696 (inv)	0.002
PfEMP1 48 Ex2	PFD0995ce2s1	PfEMP1 - Intracellular	0.696 (inv)	0.002
PfEMP1 3 Ex2	PF08_0141e2s1	PfEMP1 - Intracellular	0.694 (inv)	0.002
PfEMP1 11.1	PFF0020c_1	PfEMP1 - Extracellular - non-CD36-binding	0.692 (inv)	0.002
PfEMP1 23 Ex2	PF07_0048_ATS	PfEMP1 - Intracellular	0.689 (inv)	0.002
		**Severe malarial anemia**		
PfEMP1 18.1	MAL7P1.50_1	PfEMP1 - Extracellular - CD36-binding	0.698	0.021
PfEMP1 29.2	PF08_0106_2	PfEMP1 - Extracellular - CD36-binding	0.323	0.043
AMA1 FMP2.1 D	AMA1 FMP2.1 Vaccine Antigen (Dutta) 0.146mg/ml	AMA1	0.321	0.041
AMA1_125.1	AMA1_125.1	AMA1	0.319	0.039
STEVOR 2	MAL7P1.227	STEVOR	0.313	0.032
PfEMP1 46.2	PFD0630c_2	PfEMP1 - Extracellular - CD36-binding	0.304	0.025
PfEMP1 50.1	PFD1015c_1	PfEMP1 - Extracellular - CD36-binding	0.302	0.024
PfEMP1 44.2	PFD0615c_2	PfEMP1 - Extracellular - CD36-binding	0.296	0.020
PfEMP1 45.2	PFD0625c_2	PfEMP1 - Extracellular - CD36-binding	0.294	0.014
PfEMP1 10.2	PFE1640w_3	PfEMP1 - Extracellular - non-CD36-binding	0.270	0.008
RH5_1.1	RH5_1.1	RH5	0.758 (inv)	0.003
PfEMP1 27.2	PF07_0139_2	PfEMP1 - Extracellular - CD36-binding	0.746 (inv)	0.004
MSP 175-6	MSP 175-6	MSP1-19	0.742 (inv)	0.006
PfEMP1 10.2	PFE1640w_3	PfEMP1 - Extracellular - non-CD36-binding	0.730 (inv)	0.008
PfEMP1 45.2	PFD0625c_2	PfEMP1 - Extracellular - CD36-binding	0.706 (inv)	0.014
PfEMP1 44.2	PFD0615c_2	PfEMP1 - Extracellular - CD36-binding	0.704 (inv)	0.020
PfEMP1 50.1	PFD1015c_1	PfEMP1 - Extracellular - CD36-binding	0.698 (inv)	0.024
PfEMP1 46.2	PFD0630c_2	PfEMP1 - Extracellular - CD36-binding	0.696 (inv)	0.025
STEVOR 2	MAL7P1.227	STEVOR	0.688 (inv)	0.032
AMA1_125.1	AMA1_125.1	AMA1	0.681 (inv)	0.039

**Cerebral malaria and severe malarial anemia**				
AMA1_26.1	AMA1_26.1	AMA1	0.762	0.036
AMA1_44.1	AMA1_44.1	AMA1	0.758	0.041
AMA1_186.1	AMA1_186.1	AMA1	0.750	0.045
PfEMP1 58.1	PFL1955w_1	PfEMP1 - Extracellular - CD36-binding	0.296	0.048
PfEMP1 9.5	PFD1235w_5	PfEMP1 - Extracellular - non-CD36-binding	0.254	0.031
PfEMP1 4.3	PF11_0008e1s1	PfEMP1 - Extracellular - non-CD36-binding	0.254	0.044
PfEMP1 10.1	PFE1640w_2	PfEMP1 - Extracellular - non-CD36-binding	0.254	0.048
PfEMP1 24.2	PF07_0049_2	PfEMP1 - Extracellular - CD36-binding	0.254	0.050
PfEMP1 16.3	MAL6P1252_3	PfEMP1 - Extracellular - CD36-binding	0.250	0.045
PfEMP1 30.2	PF08_0107e1s1	PfEMP1 - Extracellular - CD36-binding	0.246	0.013
PfEMP1 26.1	PF07_0051_1	PfEMP1 - Extracellular - CD36-binding	0.931 (inv)	0.001
PfEMP1 36.2	PF13_0364_2	PfEMP1 - Extracellular - CD36-binding	0.931 (inv)	0.001
PfEMP1 50.1	PFD1015c_1	PfEMP1 - Extracellular - CD36-binding	0.908 (inv)	0.001
PfEMP1 27.1	PF07_0139_1	PfEMP1 - Extracellular - CD36-binding	0.900 (inv)	0.001
PfEMP1 7 tag	PFA0015c	DBLalpha tag Group A	0.892 (inv)	0.001
PfEMP1 58.2	PFL1955w_2	PfEMP1 - Extracellular - CD36-binding	0.885 (inv)	0.002
PfEMP1 55.1	PFL0020w_3	PfEMP1 - Extracellular - CD36-binding	0.885 (inv)	0.002
PfEMP1 28.1	PF08_0103_1	PfEMP1 - Extracellular - CD36-binding	0.877 (inv)	0.002
PfEMP1 54.1	PFL0005w_1	PfEMP1 - Extracellular - CD36-binding	0.877 (inv)	0.002
PfEMP1 22.1	MAL8P1220_2	PfEMP1 - Extracellular - CD36-binding	0.877 (inv)	0.003
PfEMP1 10.5	PFE1640w_6	PfEMP1 - Extracellular - non-CD36-binding	0.877 (inv)	0.003
PfEMP1 37.2	PFA0005w_2	PfEMP1 - Extracellular - CD36-binding	0.869 (inv)	0.003
PfEMP1 29.1	PF08_0106_1	PfEMP1 - Extracellular - CD36-binding	0.865 (inv)	0.002
PfEMP1 48.3	PFD0995ce1s1	PfEMP1 - Extracellular - CD36-binding	0.862 (inv)	0.003
PfEMP1 29.2	PF08_0106_2	PfEMP1 - Extracellular - CD36-binding	0.862 (inv)	0.004
PfEMP1 48.2	PFD0995c_2	PfEMP1 - Extracellular - CD36-binding	0.846 (inv)	0.006
PfEMP1 45.1	PFD0625c_1	PfEMP1 - Extracellular - CD36-binding	0.838 (inv)	0.007
PfEMP1 27.2	PF07_0139_2	PfEMP1 - Extracellular - CD36-binding	0.831 (inv)	0.007
PfEMP1 43.1	PFD0005w_2	PfEMP1 - Extracellular - CD36-binding	0.831 (inv)	0.008
PfEMP1 3.3	PF08_0141_4	PfEMP1 - Extracellular - non-CD36-binding	0.827 (inv)	0.009
PfEMP1 59.1	PFL1960w_1	PfEMP1 - Extracellular - CD36-binding	0.823 (inv)	0.008
PfEMP1 25.2	PF07_0050_2	PfEMP1 - Extracellular - CD36-binding	0.823 (inv)	0.008
STEVOR 2	MAL7P1.227	STEVOR	0.823 (inv)	0.008
PfEMP1 57.2	PFL1950w_2	PfEMP1 - Extracellular - CD36-binding	0.819 (inv)	0.004
PfEMP1 48.4	PFD0995ce1s2	PfEMP1 - Extracellular - CD36-binding	0.815 (inv)	0.007
PfEMP1 33.2	PF10_0406_2	PfEMP1 - Extracellular - CD36-binding	0.815 (inv)	0.010
PfEMP1 3.2	PF08_0141_3	PfEMP1 - Extracellular - non-CD36-binding	0.815 (inv)	0.010
PfEMP1 42.1	PFC1120c_1	PfEMP1 - Extracellular - CD36-binding	0.815 (inv)	0.010
PfEMP1 33.1	PF10_0406_1	PfEMP1 - Extracellular - CD36-binding	0.815 (inv)	0.012
PfEMP1 35.2	PF13_0001_2	PfEMP1 - Extracellular - CD36-binding	0.812 (inv)	0.012
PfEMP1 2.2	PF08_0140_2	PfEMP1 - Extracellular - non-CD36-binding	0.808 (inv)	0.014
PfEMP1 49.1	PFD1005c_1	PfEMP1 - Extracellular - CD36-binding	0.808 (inv)	0.014
PfEMP1 39.2	PFB0010w_2	PfEMP1 - Extracellular - CD36-binding	0.800 (inv)	0.014
PfEMP1 44.2	PFD0615c_2	PfEMP1 - Extracellular - CD36-binding	0.800 (inv)	0.017
PfEMP1 17.2	MAL6P14_2	PfEMP1 - Extracellular - CD36-binding	0.796 (inv)	0.016
PfEMP1 5.2	PF11_0521_3	PfEMP1 - Extracellular - non-CD36-binding	0.796 (inv)	0.018
PfEMP1 6.4	PF13_0003_4	PfEMP1 - Extracellular - non-CD36-binding	0.792 (inv)	0.016
PfEMP1 20.1	MAL7P150_2	PfEMP1 - Extracellular - CD36-binding	0.792 (inv)	0.018

PfEMP1 5.1	PF11_0521_2	PfEMP1 - Extracellular - non-CD36-binding	0.792 (inv)	0.018
PfEMP1 34.2	PF11_0007_2	PfEMP1 - Extracellular - CD36-binding	0.792 (inv)	0.020
PfEMP1 21.1	MAL7P155_1	PfEMP1 - Extracellular - CD36-binding	0.792 (inv)	0.020
PfEMP1 25.1	PF07_0050_1	PfEMP1 - Extracellular - CD36-binding	0.785 (inv)	0.020
PfEMP1 59.2	PFL1960w_2	PfEMP1 - Extracellular - CD36-binding	0.785 (inv)	0.022
PfEMP1 9.4	PFD1235w_4	PfEMP1 - Extracellular - non-CD36-binding	0.777 (inv)	0.026
PfEMP1 23.1	PF07_0048_2	PfEMP1 - Extracellular - CD36-binding	0.769 (inv)	0.029
PfEMP1 14.1	PFL0030c_1	PfEMP1 - Extracellular - non-CD36-binding	0.769 (inv)	0.030
PfEMP1 15.1	MAL6P11_1	PfEMP1 - Extracellular - CD36-binding	0.769 (inv)	0.032
PfEMP1 1.2	MAL6P1316_2	PfEMP1 - Extracellular - non-CD36-binding	0.765 (inv)	0.028
PfEMP1 11.1	PFF0020c_1	PfEMP1 - Extracellular - non-CD36-binding	0.765 (inv)	0.035
PfEMP1 30.1	PF08_0107_2	PfEMP1 - Extracellular - CD36-binding	0.762 (inv)	0.015
PfEMP1 3.4	PF08_0141e1s1	PfEMP1 - Extracellular - non-CD36-binding	0.762 (inv)	0.028
PfEMP1 16.1	MAL6P1252_1	PfEMP1 - Extracellular - CD36-binding	0.762 (inv)	0.036
PfEMP1 32.1	PF10_0001_2	PfEMP1 - Extracellular - CD36-binding	0.762 (inv)	0.036
PfEMP1 52.1	PFE0005w_2	PfEMP1 - Extracellular - CD36-binding	0.762 (inv)	0.037
PfEMP1 30.2	PF08_0107e1s1	PfEMP1 - Extracellular - CD36-binding	0.754 (inv)	0.013
PfEMP1 9.2	PFD1235w_2	PfEMP1 - Extracellular - non-CD36-binding	0.754 (inv)	0.042
PfEMP1 19.1	MAL7P1212_1	PfEMP1 - Extracellular - CD36-binding	0.754 (inv)	0.042
PfEMP1 16.3	MAL6P1252_3	PfEMP1 - Extracellular - CD36-binding	0.750 (inv)	0.045
PfEMP1 9.5	PFD1235w_5	PfEMP1 - Extracellular - non-CD36-binding	0.746 (inv)	0.031
PfEMP1 4.3	PF11_0008e1s1	PfEMP1 - Extracellular - non-CD36-binding	0.746 (inv)	0.044
PfEMP1 10.1	PFE1640w_2	PfEMP1 - Extracellular - non-CD36-binding	0.746 (inv)	0.048
PfEMP1 24.2	PF07_0049_2	PfEMP1 - Extracellular - CD36-binding	0.746 (inv)	0.050
PfEMP1 58.1	PFL1955w_1	PfEMP1 - Extracellular - CD36-binding	0.704 (inv)	0.048

Table S5. Area under the curve for each feature count for leave one out cross-validation (LOOCV) analyses of antigens distinguishing severe malaria cases from uncomplicated malaria controls.

Feature count	Area under the curve comparison with uncomplicated malaria controls		
	Cerebral malaria	Severe malarial anemia	Both cerebral malaria and severe malarial anemia
1	0.651	0.488	0.616
2	0.617	0.790	0.586
3	0.668	0.736	0.444
4	0.648	0.708	0.616
5	0.612	0.631	0.702
6	0.587	0.583	0.576
7	0.578	0.595	0.717
8	0.532	0.746	0.737
9	0.535	0.736	0.576
10	0.577	0.690	0.636

Diagram S1

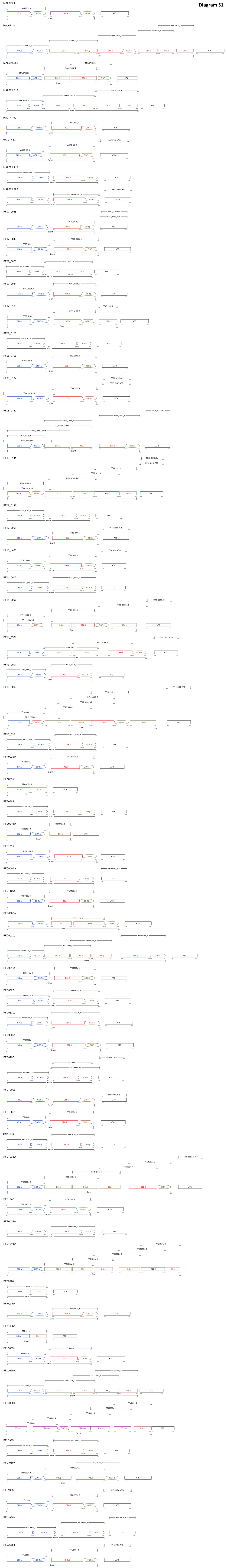
MAL6P1.1
MAL6P1.4
MAL6P1.252
MAL6P1.316
MAL7P1.50
MAL7P1.55
MAL7P1.212
MAL6P1.220
PF07_0048
PF07_0049
PF07_0050
PF07_0051
PF07_0139
PF08_0103
PF08_0106
PF08_0107
PF08_0140
PF08_0141
PF08_0142
PF10_0001
PF10_0406
PF11_0007
PF11_0008
PF11_0521
PF13_0001
PF13_0003
PF13_0364
PFA0005w
PFA0015c
PFA0765c
PFB0010w
PFB1055c
PFC0005w
PFC1120c
PFD0005w
PFD0020c
PFD0615c
PFD0625c
PFD0630c
PFD0635c
PFD0995c
PFD1000c
PFD1005c
PFD1015c
PFD1235w
PFD1245c
PFE0005w
PFE1640w
PFF0020c
PFI0005w
PFI1820w
PFL0005w
PFL0020w
PFL0030c
PFL0935c
PFL1950w
PFL1955w
PFL1960w
PFL2665c
